# Glutathione-depleting photosensitizers for tumor-specific imaging and pyroptosis-driven photodynamic therapy

**DOI:** 10.7150/thno.124154

**Published:** 2026-01-01

**Authors:** Hui Yang, Yifei Yang, Zhengyumeng Zhu, Pei Chen, Jucai Gao, Yajun Lin, Qiang Wang, Fang Hu

**Affiliations:** 1 Guangdong Provincial Key Laboratory of Medial Image Processing, Biomaterials Research Center, School of Biomedical Engineering, Southern Medical University, Guangzhou 510515, China.; 2 School of Medical Information Engineering, Gannan Medical University, Ganzhou 341000, China.; 3 Beijing National Laboratory for Molecular Sciences, Beijing 100084, China.

**Keywords:** controllable pyroptosis, GSH activation, GSH depletion, photodynamic therapy, aggregation-induced emission

## Abstract

**Rationale:** Selective initiation of pyroptosis in malignant cells can amplify the immunological benefits of photodynamic therapy (PDT), but conventional photosensitizers (PSs) often lack tumor specificity and require complex subcellular targeting motifs. Here we describe a glutathione (GSH)-responsive PDT platform based on PSs that integrate fluorescence turn-on, GSH depletion, and restoration of reactive oxygen species (ROS) generation into a single molecular design.

**Methods:** GSH-activated photosensitizers MTP-NO_2_ and NTP-NO_2_ were synthesized based on donor-acceptor structure, with their GSH-triggered activation, GSH depletion, ROS restoration, and caspase-1/GSDMD-mediated pyroptosis systematically demonstrated in 4T1 cells, while tumor accumulation, biodistribution, *in vivo* activation, and photodynamic antitumor efficacy of PSs nanoparticles were comprehensively assessed in 4T1 tumor-bearing mice through fluorescence imaging and immunohistochemical analyses.

**Results:** Among a library of donor-acceptor scaffolds, the π-extended acene derivative NTP-NO_2_, equipped with a para-dinitrophenoxybenzyl pyridinium quencher, exhibited strong optical activation and ROS production upon reaction with elevated GSH in tumor cells. This dual action, antioxidant depletion and ROS restoration, triggered caspase-1/gasdermin-D-mediated pyroptosis, IL-1β/IL-18 release, and robust immunogenic cell death. Nanoparticle delivery of NTP-NO_2_ achieved high tumor accumulation, precise imaging, and pronounced antitumor efficacy *in vivo*.

**Conclusion:** By exploiting tumor GSH overexpression-activated photodynamic therapy, the NTP-NO_2_ depletes GSH and promotes caspase-1/GSDMD pathway to trigger robust pyroptosis, eliciting inflammatory/immune responses both *in vitro* and *in vivo*. This chemically defined approach provides a PS design that unites selective activation, immune-stimulatory cell death, and precise photodynamic tumor ablation.

## Introduction

Pyroptosis is a novel form of programmed cell death characterized by rapid membrane rupture, cell swelling, and the release of pro-inflammatory cellular contents, which triggers a strong inflammatory response 1, 2. This process is mediated by the gasdermin protein family, particularly Gasdermin-D (GSDMD), which forms membrane pores after activation by caspase proteins, such as caspase-1 3-5. These cleaved fragments of GSDMD, including the N-terminal fragment, bind to phospholipids in the cell membrane, leading to cell swelling, cytoplasmic efflux, and membrane rupture 6. Additionally, active caspase-1 promotes the maturation of interleukin-1β (IL-1β) and interleukin-18 (IL-18), which then translocate through the membrane pores into the extracellular space 7, 8. These mature cytokines recruit inflammatory cells, initiating an inflammatory cascade and enhancing the effect of immunogenic cell death (ICD) 9-11. Wang et al. demonstrated that pyroptosis in only about 15% of tumor cells is sufficient to eradicate the entire 4T1 breast tumor xenograft by eliciting a robust antitumor immune response 10.

Effective pharmacological agents that can induce pyroptosis remain limited in cancer treatment 12. Chemotherapeutic agents like doxorubicin require high doses to trigger pyroptosis, but such concentrations can be harmful 13. Other strategies, including electrical stimulation and radiation therapy, have also been investigated for inducing pyroptosis in tumor cells 14, 15. However, these methods face significant challenges due to the lack of controllability 16, 17. Pyroptosis can trigger a strong immune response 10, 18, 19. Uncontrolled pyroptosis can lead to nerve damage, inflammatory diseases, and metabolic disorders, potentially progressing to various conditions such as diabetes, cardiovascular diseases, acute kidney injury, and neurological disorder 18, 20-23. Therefore, it is essential to develop an accurate and controllable strategy to achieve the on-demand initiation of pyroptosis in cancer cells while protecting normal tissues 24.

In recent years, a variety of synergistic treatment strategies have been developed to enhance antitumor efficacy 25. For instance, Long et al developed a novel nanomedicine by loading cinobufagin (CS-1) into Prussian blue nanoparticles (PB NPs), which combined drug delivery and photothermal therapy to induce pyroptosis for the treatment of triple-negative breast cancer 26. However, such approaches often rely on specially designed materials to construct nanodelivery systems for efficient drug enrichment at tumor sites. To simplify material preparation, photosensitizers have been widely adopted in photodynamic therapy for cancer treatment 27. Photodynamic therapy (PDT), a non-invasive treatment, has gained widespread application in clinical oncology owing to its high spatiotemporal precision 28-31. This technique operates through light-mediated activation of photosensitizers (PSs) to generate reactive oxygen species (ROS), thereby inducing tumor cell destruction 32-34. Studies also demonstrate that supraphysiological ROS levels can serve as inducers of pyroptosis 20, 35. However, achieving a high proportion of PDT-induced pyroptosis typically requires PS accumulation in specific organelles, such as the cell membrane, mitochondria, or early endosomes 36-40. This requirement complicates PS design 27. Additionally, the poor selectivity of conventional PSs for malignant cells and the destructive nature of apoptosis risk damaging healthy tissues during PDT 41, 42. Therefore, there is a requirement for a strategy that enhances PDT-induced pyroptosis without relying on organelle-specific targeting, thus simplifies PS design, and ensures selective tumor cell destruction to improve therapeutic efficacy and safety.

**Scheme 1:**
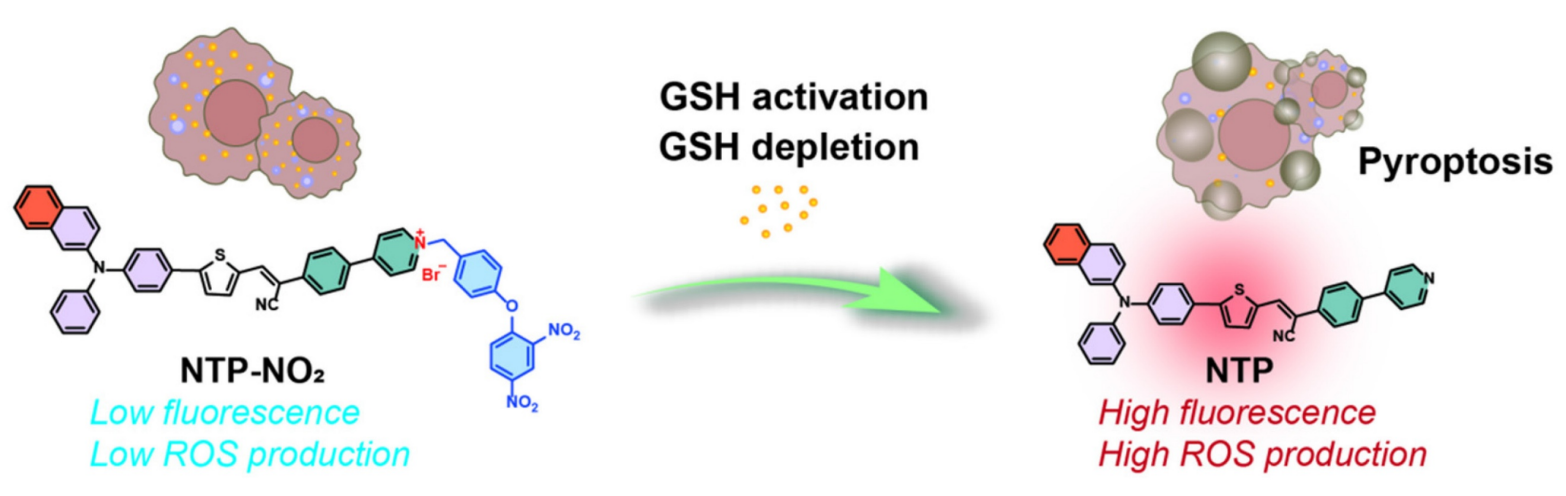
Mechanistic illustration of GSH depletion effect promoting pyroptosis.

To address these challenges, we developed a novel strategy leveraging glutathione (GSH), an overexpressed antioxidant in cancer cells, to achieve selective and controlled PDT-induced pyroptosis. High GSH levels in tumor cells scavenge ROS, reducing PDT efficacy 43-45. We designed two GSH-activated PSs, MTP-NO_2_ and NTP-NO_2_, which remain inactive in normal tissues but are selectively activated by GSH in tumor cells. Upon activation, these PSs deplete GSH and restore ROS production under laser irradiation, triggering pyroptosis via the caspase-1/GSDMD pathway (Scheme 1). Without GSH depletion, the pyroptosis was apparently reduced by the PDT of “always-on” NTP, which proves that GSH depletion is a vital controllable factor for PDT-induced pyroptosis. This process promotes the release of IL-1β and IL-18, amplifying ICD through inflammatory cell recruitment. By eliminating the need for organelle-specific targeting, our GSH-activated PSs simplify PS design, enhance tumor selectivity, and minimize damage to normal tissues, establishing a precise and safe approach for pyroptosis-based cancer therapy.

## Results and Discussion

### Molecular design and photophysical properties

**Figure 1:**
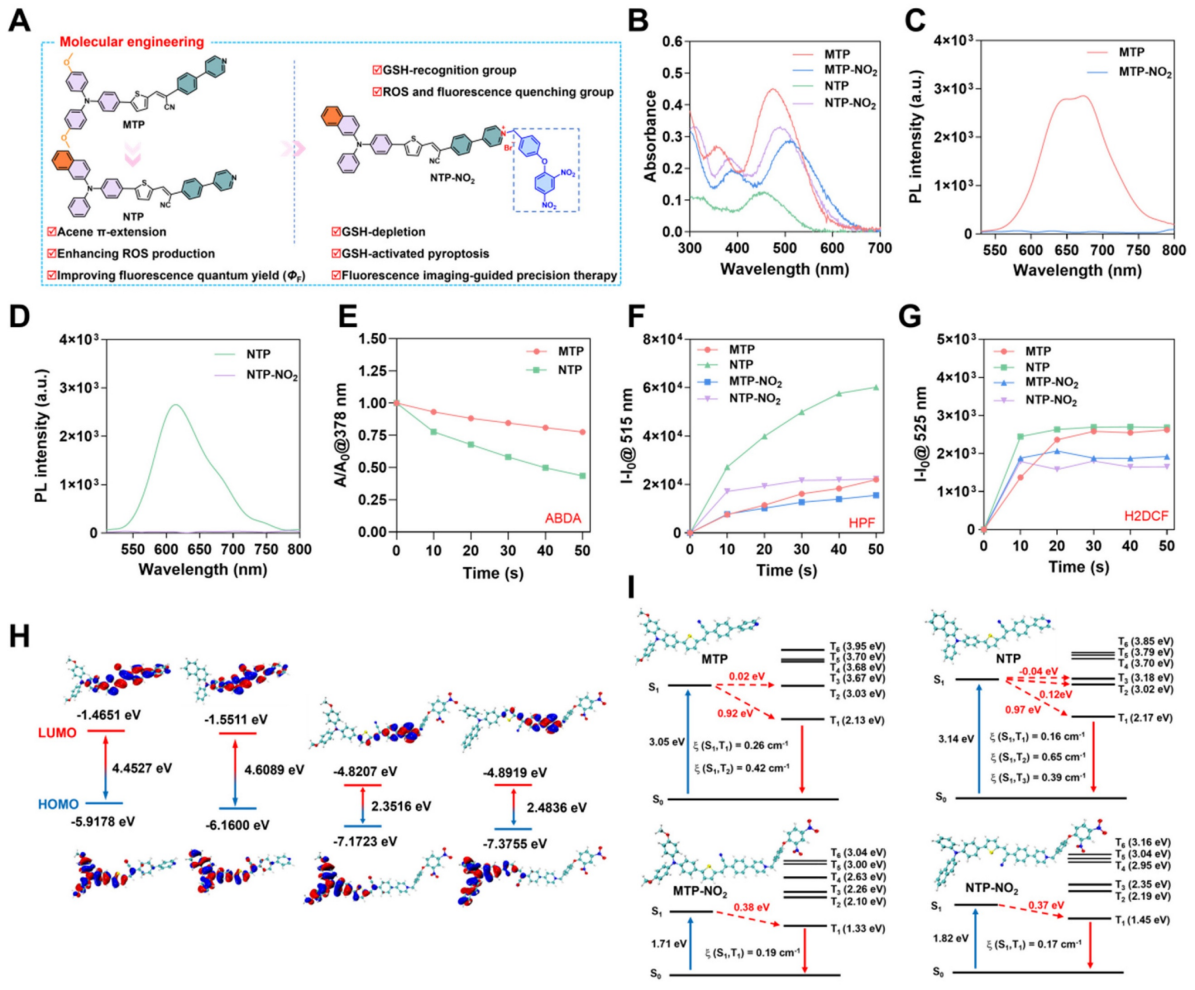
Design and characterization of PSs. (A) Schematic diagram of molecular design mechanism. (B) UV-vis absorption spectra and (C, D) fluorescence emission spectra of MTP, NTP, MTP-NO_2_, and NTP-NO_2_ in DMSO : H_2_O = 1 : 99 (v/v). (E) Degradation rate of ABDA under 530 nm laser irradiation (100 mW/cm^2^) in the presence of MTP and NTP. Plot of ΔFl. (F-F_0_) for (F) HPF at 515 nm and (G) H2DCF at 525 nm upon light irradiation (530 nm, 100 mW/cm^2^) for different time intervals in the presence of MTP, NTP, MTP-NO_2_, or NTP-NO_2_. (H) The HOMO-LUMO distributions for MTP, NTP, MTP-NO_2_, and NTP-NO_2_. (I) Using TD-DFT, an investigation was conducted on the singlet and triplet orbitals, as well as the spin-orbital coupling values, of MTP, NTP, MTP-NO_2_, and NTP-NO_2_.

MTP possesses a D-π-A structure, featuring near-infrared fluorescence emission and ROS generation, with methoxy triphenylamine serving as the electron donor, a thiophene unit as the π bridge and electron donor, and 2-(4-(pyridin-4-yl)phenyl)acetonitrile as the electron acceptor. NTP is a potentially-improved PS by replacing the methoxy with π-extending acene 31. Scheme S1 illustrates the synthetic routes of the target molecules. The compounds MTP and NTP were constructed by employing Suzuki, Buchwald-Hartwig, and Knoevenagel condensation reactions. To achieve GSH activation and consumption, para-dinitrophenoxybenzyl pyridinium moiety was introduced into MTP and NTP, yielding MTP-NO_2_ and NTP-NO_2_ (Figure 1A). The synthesis was proceeded *via* the pyridine halogenation reactions of MTP and NTP with 1-(4-(bromomethyl)phenoxy)-2,4-dinitrobenzene. The chemical structures were well characterized by ^1^H NMR, ^13^C NMR, and mass spectrometry (Figure S1-14). The characteristic FT-IR peaks of MTP, NTP, MTP-NO_2_, and NTP-NO_2_ were analyzed (Figure S15).

The photophysical properties of MTP, NTP, MTP-NO_2_, and NTP-NO_2_ were firstly investigated. As depicted in Figure 1B-D, MTP, NTP, MTP-NO_2_, and NTP-NO_2_ exhibit maximum absorption at 474 nm, 456 nm, 510 nm, and 490 nm, respectively in water. The corresponding fluorescence emission peaks for MTP and NTP are located at 673 nm and 614 nm, respectively. The para-dinitrophenoxybenzyl pyridinium moiety leads to stronger intramolecular charge transfer; consequently, the fluorescence intensity of MTP-NO_2_ and NTP-NO_2_ shows weak emission in aqueous solution, decreasing by 68.0 and 85.1 times, respectively. Using 4-(dicyanomethylene)-2-methyl-6-(4-dimethylaminostyryl)-4H-pyran (DCM) as a referenece, the *Φ*_F_ of MTP and NTP in water were measured to be 0.38% and 11.4%, respectively (Figure S16). Furthermore, the aggregation-induced emission (AIE) features of MTP and NTP were confirmed by monitoring the variations in their fluorescence intensities within DMSO/water mixtures of differing water fractions. (Figure S17).

The photosensitization efficiency of MTP, NTP, MTP-NO_2_, and NTP-NO_2_ were then estimated. To detect the photosensitizing capacity of MTP and NTP, the singlet oxygen (^1^O_2_) probe 9,10-anthracenyl-bis(methylene)dimalonic acid (ABDA) was utilized (Figure 1E; Figure S18). The Hydroxyl radical (•OH) production capacities were assessed using the fluorescent indicator Hydroxyphenyl Fluorescein (HPF) (Figure 1F; Figure S19) and the total ROS production capacities were measured by 2′,7′-dichlorodihydrofluorescein (H2DCF) (Figure 1G; Figure S20). As summarized in Table 1, compared with MTP, the ^1^O_2_ production capacity, ROS production capability, and •OH production capability of NTP increase to 2.46, 1.11, and 2.73 times of MTP. In addition, the *Φ*_F_ of NTP is 30 times of MTP. Overall, the π extension through acene enlargement can not only enhance photosensitizing efficiency but also increase the *Φ*_F_. Importantly, both MTP and NTP exhibit significantly decreased photosensitizing efficiency following the attachment of the para-dinitrophenoxybenzyl pyridinium moiety. This suggests that the para-dinitrophenoxybenzyl pyridinium moiety functions not only as a fluorescence quenching group but also as an inhibitor of ROS production, which can realize fluorescence and photosensitization turn-on upon GSH activation and enable cancer cell selectivity.

To investigate the mechanism of ROS quenching and the enhancement of ROS production, Gaussian calculation was carried out. The distributions and orbitals of the highest occupied molecular orbital (HOMO) and the lowest unoccupied molecular orbital (LUMO) are presented in Figure 1H. Compared to MTP and NTP, the energy gaps between the HOMO and LUMO orbitals of MTP-NO_2_ and NTP-NO_2_ are all reduced, resulting in a red shift in absorption. Specifically, in MTP-NO_2_ and NTP-NO_2_, the reduced gap of HOMO-LUMO is mainly attributed to a decrease in LUMO energy levels caused by the strong electron-withdrawing effect of the pyridinium salt. To probe the singlet and triplet orbitals of MTP, MTP-NO_2_, NTP, and NTP-NO_2_, time-dependent density functional theory (TD-DFT) calculations were undertaken. Figure 1I illustrates the S_n_ and T_n_ (n = 1-6) orbital energy levels. The first excited singlet state (S_1_) of NTP increases in energy level relative to that of MTP, bringing it closer to the higher triplet excited states (T_n_, n = 2-6). Concurrently, the intersystem crossing (ISC) process involved in ROS generation was facilitated, promoted by the movement of the higher triplet excited states (T_n_, n = 2-6) of NTP closer to the ground state. Furthermore, an energy gap of less than 0.3 eV between the S_1_ and T_n_ states leads to an enhanced ISC efficiency. TD-DFT computational results reveal that only the S_1_ to T_2_ channel is favorable in MTP, whereas multiple channels from S_1_ to T_2_ and T_3_ are favorable in NTP. Consequently, the presence of these additional efficient singlet-to-triplet channels leads to the enhanced ROS production capacity of NTP. After modification with the para-dinitrophenoxybenzyl pyridinium moiety, no S_1_ to T_2_ channel is favorable in either MTP-NO_2_ or NTP-NO_2_, resulting in blocked ROS generation.

### Preparation and characterization of nanoparticles and their responsiveness to GSH

**Figure 2:**
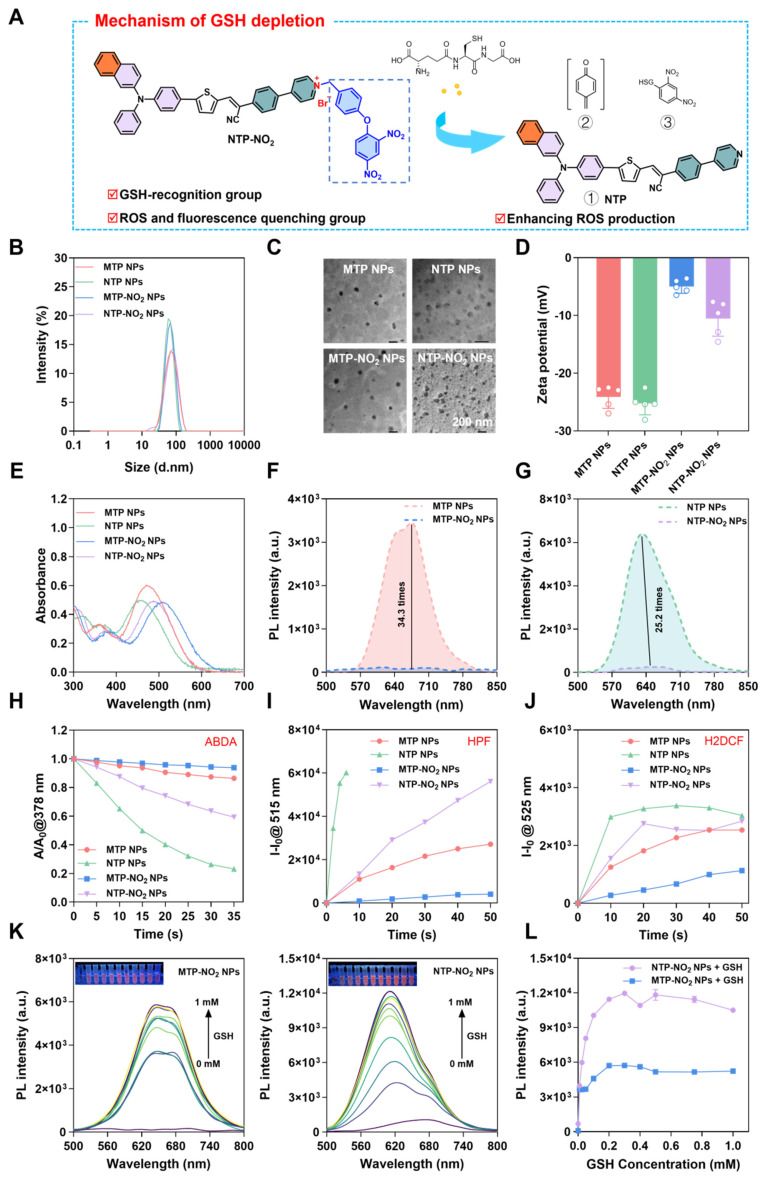
Preparation, characterization, and GSH-responsive testing of NPs. (A) Schematic of GSH depletion mechanism. (B) DLS size distribution and (C) TEM image of NPs. (D) Zeta potential of NPs. (E) UV-vis absorption spectra of NPs. (F, G) Fluorescence spectra of NPs. (H) Decomposition rates of ABDA with NPs (10 µg/mL) under 530 nm laser irradiation (100 mW/cm^2^) at varied durations. (I) Plot of ΔFl. (F-F_0_) for HPF at 515 nm upon light irradiation (530 nm, 100 mW/cm^2^) for different time intervals in the presence of NPs (10 µg/mL). (J) Plot of ΔFl. (F-F_0_) for H2DCF at 525 nm upon light irradiation (530 nm, 100 mW/cm^2^) for different time intervals in the presence of NPs (10 µg/mL). (K) Emission spectra of MTP-NO_2_ NPs and NTP-NO_2_ NPs after incubation with GSH (0-1.0 mM) in PBS/DMSO (v/v = 3/1). Inset: Fluorescence under 365 nm UV lamp post GSH incubation (the concentration of NPs = 10 µg/mL). (L) Fluorescence intensity of MTP-NO_2_ NPs and NTP-NO_2_ NPs after incubation with varying GSH concentrations (n = 3). Error bars represent the mean ± SD.

The response mechanism of MTP-NO_2_ and NTP-NO_2_ for GSH is proposed in Figure 2A. The mechanism was confirmed by mass spectra of the reaction mixtures. As shown in Figure S21-26, after incubation with GSH, the molecular ion peak of 592.2063 and 582.2006 were observed, accord with the theoretical molecular mass of [MTP + H^+^] = 592.1980 and [NTP + H^+^] = 582.1926, respectively. Molecules in nanoparticle-loaded form can be applied more flexibly *in vivo*. Therefore, encapsulation with DSPE-PEG2000 was used to prepare the four PSs into water-dispersible nanoparticles (NPs) (Figure S27A). The four types of nanoparticles, MTP NPs, NTP NPs, MTP-NO_2_ NPs, and NTP-NO_2_ NPs, exhibited an average hydrodynamic diameter of approximately 70 nm, as measured by dynamic light scattering (DLS) (Figure 2B). This size was corroborated by transmission electron microscopy (TEM) images (Figure 2C). Energy-dispersive X-ray spectroscopy (EDS) analysis revealed that the nanoparticles displayed elements C, N, O, P, and S. Additionally, the MTP-NO_2_ NPs and NTP-NO_2_ NPs groups contained Br element (Figure S28).The zeta potentials of MTP NPs, NTP NPs, MTP-NO_2_ NPs and NTP-NO_2_ NPs were also measured and they were -24.11 ± 1.98, -25.24 ± 1.98, -4.98 ± 1.18, -10.54 ± 3.05 mV, respectively (Figure 2D). The stability of MTP NPs, NTP NPs, MTP-NO_2_ NPs, and NTP-NO_2_ NPs were measured by continually monitoring the particle size and PDI for 6 days in water and the results show that their particle size and PDI were relatively constant (Figure S27B-E). And their size was constant in an aqueous solution containing 10% BSA by detecting the hydrodynamic sizes every day within 6 days (Figure S29). After incubation with GSH, neither MTP-NO_2_ NPs nor NTP-NO_2_ NPs exhibited significant changes in particle size or morphology. The zeta potentials were measured at -17.02 ± 1.03 and -15.7 ± 3.73 mV, respectively (Figure S30). MTP NPs, NTP NPs, MTP-NO_2_ NPs, and NTP-NO_2_ NPs display maximum absorption at 470 nm, 455 nm, 506 nm, and 488 nm, respectively (Figure 2E); and their fluorescence emission peaks are at 675 nm, 632 nm, 696 nm, and 665 nm. Notably, the fluorescence of MTP NPs was 34.3 times higher than that of MTP-NO_2_ NPs, and the fluorescence of NTP NPs was 25.2 times higher than that of NTP-NO_2_ NPs (Figure 2F-G). The^ 1^O_2_ production capacity (Figure 2H; Figure S31), •OH production capability (Figure 2I; Figure S32), and ROS production capability (Figure 2J; Figure S33) are ranked as follows: MTP NPs > MTP-NO_2_ NPs, NTP NPs > NTP-NO_2_ NPs. The optical behavior of all NPs is comparable to that of their aqueous aggregates.

The fluorescence changes of MTP-NO_2_ NPs and NTP-NO_2_ NPs in response to GSH were tested to verify the responsiveness. As shown in Figure S34, the fluorescence were continuously increased with increased concentrations of GSH (0.01 mM, 0.05 mM, 0.2 mM, 0.5 mM). Fluorescence saturation occurs in about 50 min at GSH concentration above 0.01 mM (fluorescence intensity variations of MTP-NO_2_ NPs and NTP-NO_2_ NPs solution at 675 nm and 633 nm, respectively). Therefore, a 50-minute response time was used for the following sensitivity and selectivity studies. As shown in Figure 2K, with the increase in GSH concentration (0 - 1 mM), the fluorescence intensity of MTP-NO_2_ NPs and NTP-NO_2_ NPs gradually increased, accompanied by an obvious color change from dark red to orange-yellow. Compared with MTP-NO_2_ NPs, an improved linearity between the fluorescence intensity of NTP-NO_2_ NPs and the concentration of GSH (0 - 0.2 mM) was observed, which is astributed to the stronger fluorescence (Figure 2L). The following section examines the probe's response mechanism to GSH. As shown in Figure S35, the addition of 0.2 mM GSH in the MTP-NO_2_ NPs and NTP-NO_2_ NPs exhibits a new absorption at 470 nm and 455 nm, respectively. Subsequently, we tested the changes in fluorescence spectra after co-incubation of MTP-NO_2_ NPs and NTP-NO_2_ NPs with various biologically relevant analytes to demonstrate the specificity of their response to GSH. As can be seen from the Figure S36, the fluorescence changes were negligible in various acids, inorganic salts, and amino acids, and although a small fluorescence change was induced in strong alkaline environments, there was almost no interference with the probes because the tumor microenvironment was acidic (pH = 6.0 - 7.0), suggesting that the MTP-NO_2_ NPs or NTP-NO_2_ NPs are highly selective for GSH.

### The GSH consumption in cells and the PDT ablation of cancer cells

**Figure 3:**
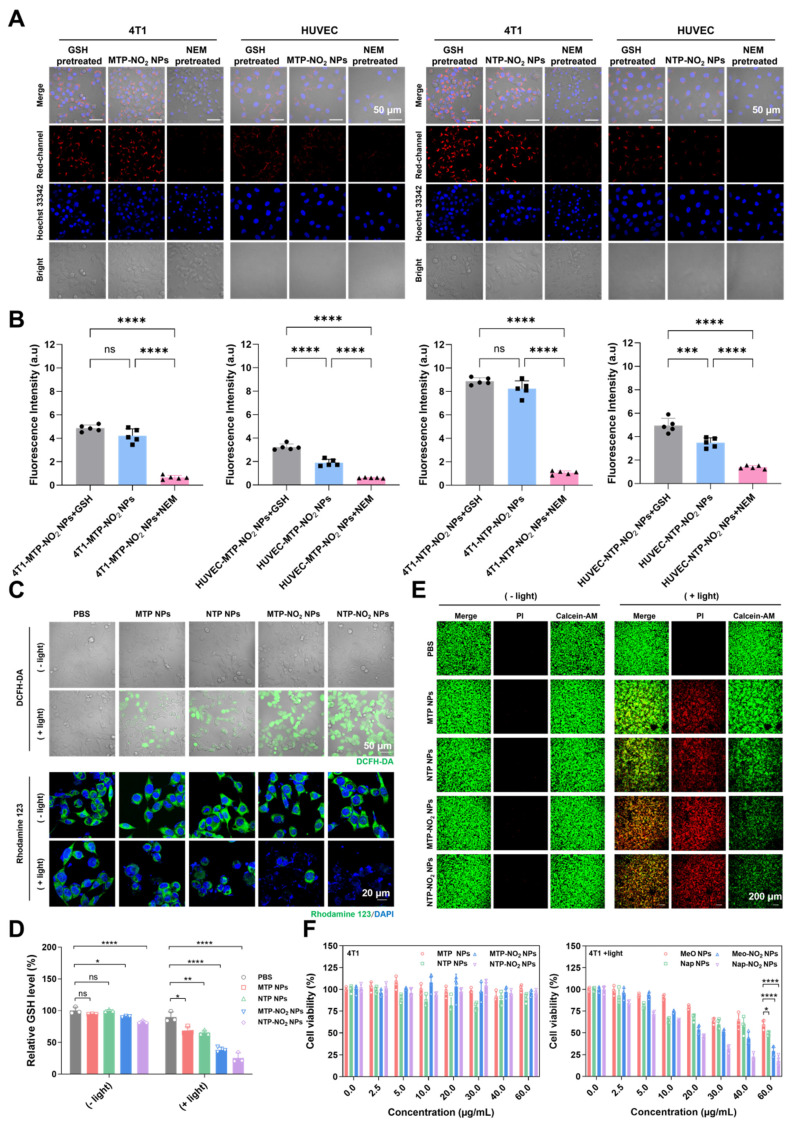
GSH-responsive activation of PDT and GSH depletion at the cellular level. (A) CLSM fluorescence intensity images and (B) Quantitative analysis of MTP-NO_2_ NPs and NTP-NO_2_ NPs in different cell types under various treatments. (C) CLSM images of 4T1 cells stained with DCFH-DA or Rhodamine 123 after treatment with PBS, MTP NPs, NTP NPs, MTP-NO_2_ NPs, or NTP-NO_2_ NPs under dark or light irradiation. (D) GSH depletion levels were measured by detection kit in cells incubated with PBS, MTP NPs, NTP NPs, MTP-NO_2_ NPs, or NTP-NO_2_ NPs under dark or light irradiation. (E) CLSM images of Calcein-AM/PI-stained 4T1 cells post-treatment with PBS, MTP NPs, NTP NPs, MTP-NO_2_ NPs, or NTP-NO_2_ NPs under dark or light irradiation. (F) Cell viability of 4T1 cells treated with MTP NPs, NTP NPs, MTP-NO_2_ NPs, or NTP-NO_2_ NPs at varying concentrations, with or without light irradiation [Irradiation intensity: 100 mW/cm^2^; irradiation duration: 10 min]. Error bars represent the mean ± SD, *p < 0.05, **p < 0.01, ***p < 0.001 and ****p < 0.0001.

To validate the responsiveness of MTP-NO_2_ NPs and NTP-NO_2_ NPs to GSH in cells. Cellular uptake was monitored by confocal laser scanning microscopy (CLSM) in the breast cancer cell line 4T1 (high GSH level). As illustrated in Figure S37, 4T1 cells showed gradually increased red fluorescence over time after incubation with the NPs, peaking at 4 h, which is sufficient for the reaction with GSH. The GSH responsiveness of MTP-NO_2_ NPs and NTP-NO_2_ NPs in cells was further confirmed. *N*-Ethylmaleimide (NEM) is a thiol-trapping agent that can reduce intracellular GSH. As shown in Figure 3A-B, in 4T1 cells, fluorescence was greatly reduced after NEM pretreatment, while the strong signals were observed in the GSH-pretreated and untreated groups, indicating that endogenous GSH was sufficient to activate the probe. The response of MTP-NO_2_ NPs and NTP-NO_2_ NPs in normal human umbilical vein endothelial cells (HUVECs, low GSH levels) was also examined to evaluate their ability to differentiate cancer cells from normal cells. Consistent with cancer cells, fluorescence in HUVECs was much weaker, likely due to lower GSH levels. However, exogenous GSH pretreatment in HUVECs led to increased fluorescence, reflecting the elevated GSH concentration. These findings collectively suggest that MTP-NO_2_ NPs and NTP-NO_2_ NPs exhibit superior specific GSH-responsive properties.

The intracellular ROS generation was tested by DCFH-DA. As shown in Figure 3C, under light irradiation (530 nm, 100 mW/cm^2^, 10 min), MTP-NO_2_ NPs and NTP-NO_2_ NPs produced stronger green fluorescence in 4T1 cells than MTP NPs and NTP NPs, indicating higher ROS generation in 4T1 cells. This result, opposite to that in aqueous solutions (NTP NPs > NTP-NO_2_ NPs, MTP NPs > MTP-NO_2_ NPs), stems not only from the intracellular GSH-triggered conversion of MTP-NO_2_ NPs and NTP-NO_2_ NPs into highly ROS-generating MTP and NTP, but also from the depletion of intracellular GSH, which avoids the elimination of ROS. Mitochondrial status was assessed using Rhodamine 123. After light exposure, NTP-NO_2_ NPs and MTP-NO_2_ NPs caused greater mitochondrial disruption than NTP NPs and MTP NPs, respectively (Figure 3C). A GSH assay kit was used to verify the depletion of intracellular GSH. Without light irradiation, MTP and NTP NPs cannot deplete intracellular GSH while MTP-NO_2_ and NTP-NO_2_ NPs showed intracellular GSH depletion ability. Upon light irradiation, the intracellular GSH depletion by MTP-NO_2_ NPs and NTP-NO_2_ NPs was significantly enhanced, due to the dual effects of dinitrophenoxybenzyl reaction and ROS generation (Figure 3D). To visualize cell killing by PS NPs, live/dead staining with Calcein-AM (green, live cells) and PI (red, dead cells) was performed. As shown in Figure 3E, all groups showed green fluorescence in the dark, indicating low toxicity. After light exposure, MTP-NO_2_ NPs caused more cell death than MTP NPs, and NTP-NO_2_ NPs showed more cell death than NTP NPs.

The cytotoxicity of PS NPs at different concentrations was further evaluated by MTT assay. Under dark conditions and at 60 µg/mL, both 4T1 and HUVEC cells maintained over 80% viability (Figure 3F; Figure S38), confirming low toxicity and good biocompatibility of the NPs. Under light irradiation (530 nm, 100 mW/cm^2^, 10 min), the phototoxicity against 4T1 cells, with half-maximal inhibitory concentration (IC50) values ranked as follows: NTP-NO_2_ NPs (12.79 µg/mL) > NTP NPs (42.56 µg/mL), and MTP-NO_2_ NPs (17.06 µg/mL) > MTP NPs (85.70 µg/mL). Notably, MTP-NO_2_ NPs and NTP-NO_2_ NPs showed no significant toxicity toward HUVECs in the dark and only minimal cytotoxicity under light conditions (IC50 > 100 µg/mL). The low GSH levels in normal cells result in minimal phototoxicity, demonstrating the tumor-selective activation and good biosafety of MTP-NO_2_ NPs and NTP-NO_2_ NPs. Conversely, the high intracellular GSH levels in 4T1 cells effectively activate MTP-NO_2_ NPs and NTP-NO_2_ NPs, enhancing their photodynamic efficacy. Unlike conventional PS Ce6, which lacks selective activation capability, it exerts PDT killing effects on both 4T1 cells and HUVECs (Figure S39). A positive correlation between PDT efficacy and intracellular GSH levels was also observed in other breast cancer cell lines (such as human breast cancer cells MDA-MB-231 and MCF7) and normal breast cells (human mammary epithelial cells MCF10A). As shown in Figure S40, MDA-MB-231 exhibited higher PDT efficacy than MCF7, consistent with its elevated intracellular GSH levels quantified by Monochlorobimane (mBCl) fluorescence analysis. This GSH-triggered activation not only promotes efficient ROS generation but also leads to GSH depletion, resulting in amplified oxidative stress and a synergistic PDT effect.

### Cancer cell pyroptosis *via* Gasdermin D

**Figure 4:**
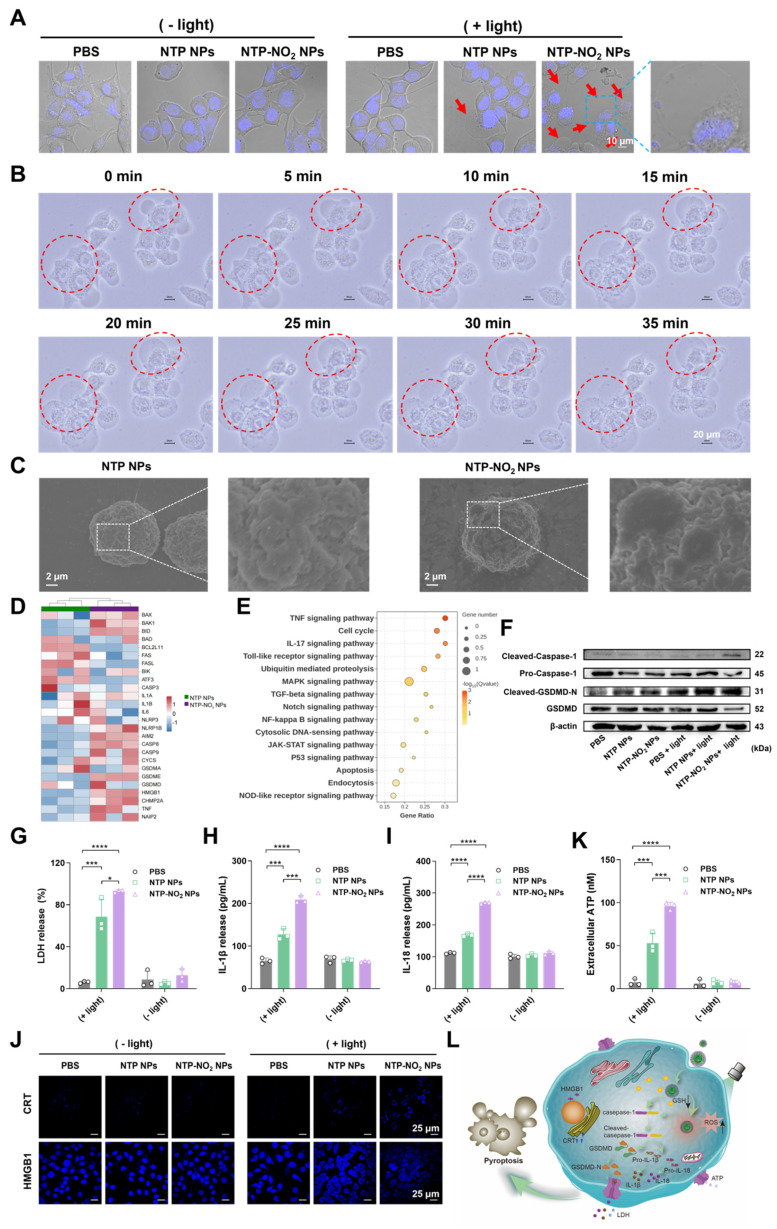
PDT-induced GSDMD-mediated pyroptosis and ICD effect. (A) Representative images observed by CLSM after incubation with NTP NPs and NTP-NO_2_ NPs. Red arrows indicate pyroptotic vesicles. (B) Time-dependent morphological changes of 4T1 cells incubated with NTP-NO_2_ NPs followed by irradiation at 100 mW/cm^2^ for 10 min. (C) SEM images after treatment with NTP NPs or NTP-NO_2_ NPs followed by illumination. Analysis of bulk RNA-seq data from 4T1 cells treated with NTP NPs and NTP-NO_2_ NPs (n = 3) reveals: (D) enrichment of apoptotic and pyroptotic target genes in the heat map, and (E) significant enrichment of inflammation and cell death-associated signaling pathways in KEGG analysis of differentially expressed genes. (F) Western blot analysis of pyroptosis-related proteins under different conditions. (G) LDH release from 4T1 cells treated with NTP NPs and NTP-NO_2_ NPs. Secretion levels of IL-1β (H) and IL-18 (I) in supernatants of 4T1 cells treated with NTP NPs and NTP-NO_2_ NPs, respectively. (J) Expression levels of CRT and HMGB1 in cells after different treatments. (K) Extracellular ATP release levels after different treatments. (L) Schematic diagram of GSH depletion and PDT-induced pyroptosis. [NPs concentration: 60 μg/mL; irradiation intensity: 100 mW/cm^2^; irradiation duration: 10 min]. Error bars represent the mean ± SD, *p < 0.05, **p < 0.01, ***p < 0.001 and ****p < 0.0001.

NTP and NTP-NO_2_ NPs were selected to study PDT-induced pyroptosis. The NPs localization was studied using commercial probes Lyso-Tracker Green, Mito-Tracker Green, and Lipid-Green in 4T1 cells. Confocal imaging showed the highest co-localization with lysosomes (Figure S41; Table S1), which are the most likely targeted organelles for NPs without special design. Hoechst 33342 staining 4T1 cells showed that nuclei remained intact without fragmentation after treatment with NTP-NO_2_ NPs with or without light irradiation, clearly distinguishing the morphology from apoptosis (Figure 4A). Treatment of 4T1 cells with NTP-NO_2_ NPs or NTP NPs followed by 530 nm laser irradiation (100 mW/cm^2^, 10 min) induced morphological changes, including cell swelling and the formation of vesicle-like protrusions (termed pyrophagosomes, indicated by red arrowheads). Much more pyrophagosomes were observed in 4T1 cells treated with NTP-NO_2_ NPs, due to NTP-NO_2_ NPs-induced depletion of intracellular GSH. As shown in Figure 4B, real-time imaging from 0 to 35 min after NTP-NO_2_ NPs treatment and light exposure exhibited gradual swelling and enlargement of the cell membrane, consistent with pyroptosis-associated morphological features. Scanning electron microscopy (SEM) imaging revealed bubble-like structures on the cell membranes in the NTP-NO_2_ NPs-treated group, indicating that the cells were undergoing pyroptosis (Figure 4C). Bulk RNA-seq confirmed that both NTP NPs and NTP-NO_2_ NPs induced apoptosis, whereas NTP-NO_2_ NPs specifically activated the inflammasome pathway and enhanced pyroptosis-related genes including NLRP1, AIM2 and GSDMD (Figure 4D).

KEGG enrichment analysis further demonstrated significant activation of inflammatory pathways (TNF, NF-kappa B) and associated pyroptosis regulators (Toll-like receptor, NOD-like receptor), collectively indicating potentiated inflammatory cell death (Figure 4E). Western blot analysis further confirmed the pyroptosis mechanism by showing activation of the N-terminal pore-forming domain GSDMD-N and increased cleaved-caspase-1 expression in 4T1 cells after NPs-PDT, consistent with pyroptosis induction. These observations align with previous reports that caspase-1 cleavage directly promotes GSDMD cleavage (Figure 4F; Figure S42). Lactate dehydrogenase (LDH) release assays demonstrated significantly higher LDH levels in the NTP-NO_2_ NPs group compared to the control and NTP NPs groups (Figure 4G). Additionally, elevated levels of the proinflammatory cytokines interleukin IL-1β and IL-18 were observed, both hallmark features of pyroptosis (Figure 4H-I), demonstrating controlled induction of pyroptosis in targeted cancer cells.

ICD is a promising strategy that stimulates the immune system 46. Among ICD types, pyroptosis triggers a stronger immune response due to its pro-inflammatory nature and rapid cell membrane rupture. The hallmark of ICD is the exposure or release of damage-associated molecular patterns, which includes calreticulin (CRT) exposed on the cell surface, high-mobility group box 1 (HMGB1) released into the cytoplasm and extracellular space 47, and adenosine triphosphate (ATP) secreted extracellularly 48. As shown in Figure 4J-K, after laser irradiation, 4T1 cells treated with NTP-NO_2_ NPs exhibited higher CRT exposure, increased HMGB1 in the cytoplasm, and significantly elevated extracellular ATP levels compared to those treated with NTP NPs, indicating stronger ICD-inducing capability of NTP-NO_2_ NPs. This is attributed to the synergistic ICD induction *via* PDT and GSH depletion-driven cascade amplification. These findings demonstrate that PDT mediated by NTP-NO_2_ NPs, along with its induction of GSH depletion, effectively promotes DAMP release by activating the GSDMD-dependent pyroptosis pathway (Figure 4L).

### Biosafety evaluation and tumor-responsive fluorescence imaging

**Figure 5:**
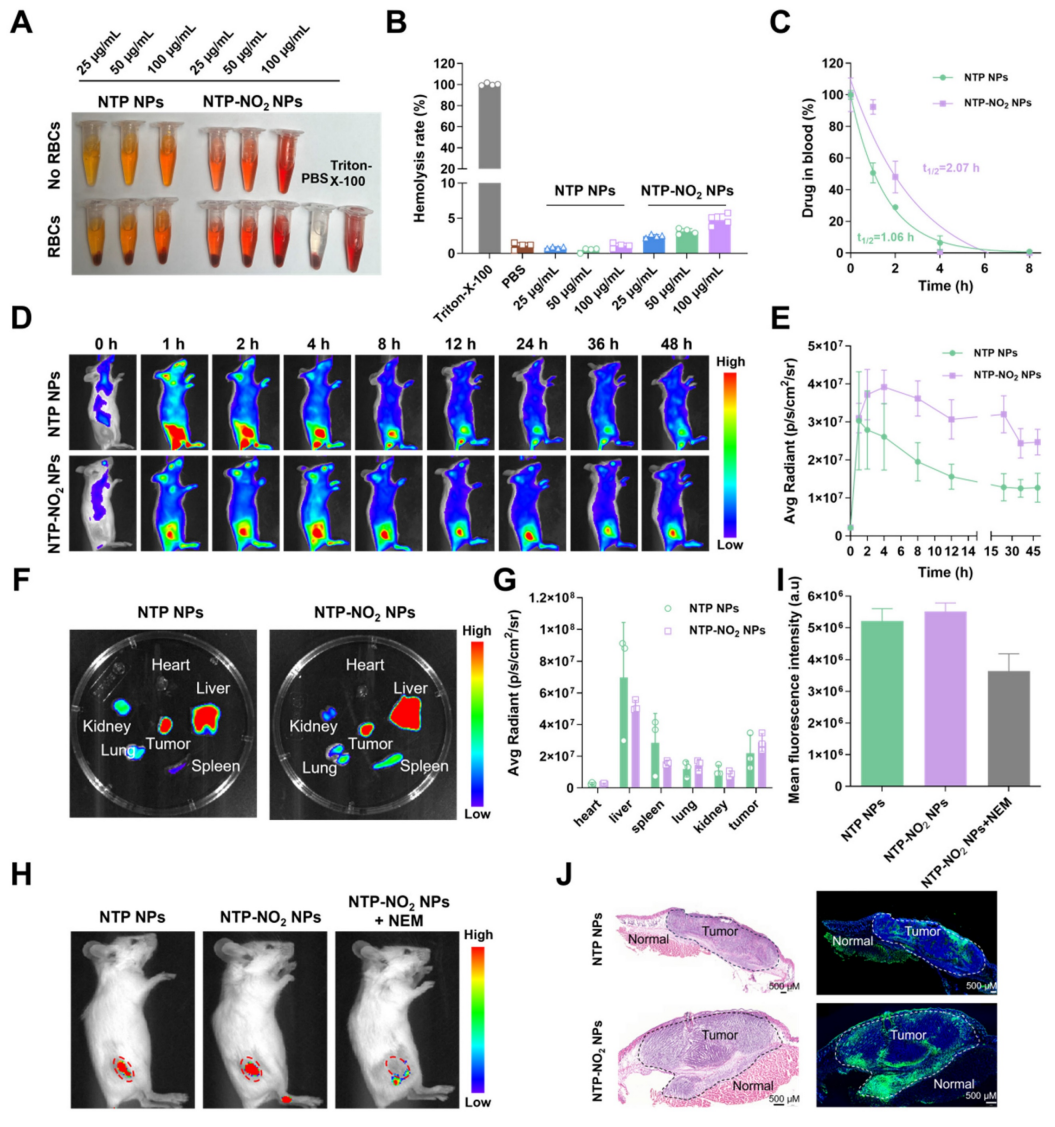
GSH activation at the animal level. (A) Assessment of hemolysis rates in red blood cells (RBCs) following treatment with NTP NPs and NTP-NO_2_ NPs (0-100 µg/mL) for 2 h at 37  °C. Triton X-100 and NPs in PBS without RBCs served as the positive and negative controls, respectively. (B) Corresponding hemolysis rates of different treatments. (C) Blood drug concentration levels at various time points (n = 3). (D) Fluorescence images capture the time-dependent biodistribution in mice after intravenous administration of NTP NPs and NTP-NO_2_ NPs over a 48 h period (0, 1, 2, 4, 8, 12, 24, 36, 48 h). (E) Tumor fluorescence intensity profiles following intravenous injection of NTP NPs and NTP-NO_2_ NPs at different time intervals (n = 3). (F) *Ex vivo* imaging of heart, liver, spleen, lungs, kidneys and tumor tissue obtained from mice 48 h after i.v. injection of NTP NPs or NTP-NO_2_ NPs. (G) Quantitative analysis of fluorescence intensity in different tissues ( n = 3). (H) *In vivo* imaging of tumor-bearing mice 4 h after tail vein injection with NTP NPs, NTP-NO_2_ NPs, and NTP-NO_2_ NPs + NEM (5 mM NEM injected intratumorally 1 h prior to NPs administration). (I) Mean tumor fluorescence intensity values corresponding to (H). (J) The fluorescence distribution of NTP NPs and NTP-NO_2_ NPs in tumor tissues and normal tissues was analyzed via H&E staining and autofluorescence imaging of tissue sections. Error bars represent the mean ± SD.

Before conducting *in vivo* experiments, the hemolytic activity of NTP NPs and NTP-NO_2_ NPs were assessed. Experimental results demonstrated that neither NTP NPs nor NTP-NO_2_ NPs induced hemolysis at a high concentration of 100 µg/mL (Figure 5A-B). Subsequently, blood pharmacokinetic analysis revealed half-lives of 1.06 h and 2.07 h for NTP NPs and NTP-NO_2_ NPs, respectively (Figure 5C). The longer circulation is possibly due to the positive charge of NTP-NO_2_ and interaction with blood proteins. The *in vivo* GSH activation was further confirmed in 4T1 tumor-bearing BALB/c mice. The positive-charged NTP-NO_2_ results in longer retention in tumor and slower decline. As shown in Figure 5D-E, both NTP NPs and NTP-NO_2_ NPs (6 mg/kg) by intravenous injection showed sustained tumor-site fluorescence. NTP NPs reached peak accumulation at 2 h, indicating efficient tumor enrichment through the enhanced permeability and retention (EPR) effect, while NTP-NO_2_ NPs reached maximum fluorescence at 4 h and delined slowly from 4 to 24 h. The longer circulation brings larger accumulation and higher fluorescence signals of NTP-NO_2_ NPs. Mice were euthanized at 48 h post-injection, and their major organs and tumors were harvested for *ex vivo* imaging. Fluorescence signals indicated predominant accumulation of NTP and NTP-NO_2_ NPs in the tumor and liver (Figure 5F-G), the NTP-NO_2_ NPs showed more accumulation in tumor and less activation in liver. To further confirm the GSH-activated imaging, NEM was intratumorally injected 1 h before intravenous administration of NTP-NO_2_ NPs (6 mg/kg). *In vivo* imaging at 4 h post-injection exhibited a significant decrease in tumor fluorescence compared to NTP-NO_2_ NPs control groups (Figure 5H-I), confirming the selective activation of NTP-NO_2_ NPs by tumor-associated GSH. Additionally, the distinct fluorescence intensity contrast between tumor and normal tissues, as confirmed by hematoxylin and eosin (H&E) staining, supported the tumor-selective activation of NTP-NO_2_ NPs. In contrast, no significant difference in fluorescence intensity was observed between tumor and normal tissues in the NTP NPs group (Figure 5J).

### Evaluation of PDT efficacy *in vivo*

**Figure 6:**
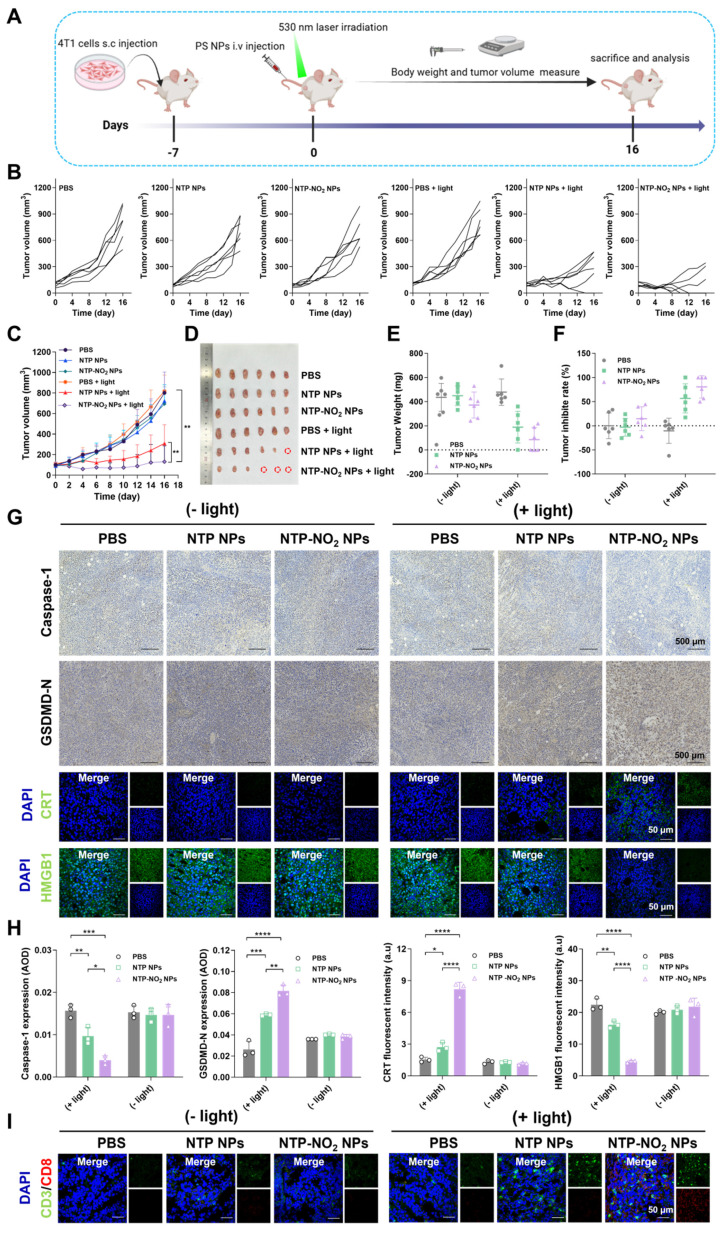
Tumor therapeutic efficacy and immune activation at the animal level. (A) Schematic of tumor implantation protocol and treatment timeline. (B) Individual tumor growth curves of mice during the 16-day treatment period (PDT parameters: 200 mW/cm^2^ irradiation for 12 min). (C) Dynamic changes in tumor volume across treatment groups (n = 6; photodynamic therapy parameters: 200 mW/cm^2^ irradiation for 12 min). (D) Excised tumor specimens from PBS, NTP NPs, NTP-NO_2_ NPs, PBS + light, NTP NPs + light and NTP-NO_2_ NPs + light groups on day 16. (E) Quantitative analysis of tumor weights across groups (n = 6). (F) Tumor inhibition rates under different treatment regimens (n = 6). (G) Immunohistochemical analysis of caspase-1 and GSDMD-N, and immunofluorescence analysis of CRT and HMGB1 in tumor tissues post-treatment. (H) Quantitative analysis of protein expression in Figure G. (I) Immunofluorescence staining analysis of CD3^+^/CD8^+^ cytotoxic T-cell maturation in tumor tissue. Error bars represent the mean ± SD, *p < 0.05, **p < 0.01, ***p < 0.001 and ****p < 0.0001.

The *in vivo* PDT efficiency and post-treatment safety of NTP NPs and NTP-NO_2_ NPs were examined in BALB/c mice bearing 4T1 tumors. In addition, the GSH-depletion-enhanced PDT efficacy was evaluated by comparing the PDT effects of NTP NPs and NTP-NO_2_ NPs. As shown in Figure 6A, 4T1 tumor-bearing mice were randomized into six groups (n = 6): (1) PBS, (2) NTP NPs, (3) NTP-NO_2_ NPs, (4) PBS + light, (5) NTP NPs + light, and (6) NTP-NO_2_ NPs + light; at a dose of 6 mg/kg. Tumor growth in the “NTP NPs”, “NTP-NO_2_ NPs”, and “PBS + light” groups increased rapidly, similar to the PBS control group. In contrast, tumor growth in the “NTP NPs + light” and “NTP-NO_2_ NPs + light” groups was obviously inhibited with some even being cleared.

The inhibitory effect in the “NTP-NO_2_ NPs + light” group was stronger than that in the “NTP NPs + light” group (Figure 6B). As shown in Figure 6C, both NTP NPs and NTP-NO_2_ NPs significantly inhibited tumor growth over 16 days, demonstrating their effective *in vivo* PDT performance. After 16 days of treatment, the implanted tumors were excised and the tumor sizes were directly compared (Figure 6D). The tumor volume and size in “NTP-NO_2_ NPs + light” group were smallest compared to all other groups (Figure 6E). The excised tumors were weighed, and the NTP-NO_2_ NPs group exhibited the lowest tumor weight, with an average inhibition rate of 85.18% (Figure 6F). To comprehensively assess the biocompatibility and antitumor efficacy of PS NPs, the major organs and tumor were stained with H&E, terminal deoxynucleotidyl transferase dUTP nick end labeling (TUNEL), and Ki67. As shown in Figure S43, H&E and TUNEL staining revealed significant tumor tissue damage in both “NTP NPs + light” and “NTP-NO_2_ NPs + light” groups, with greater damage observed in the latter. In contrast, no damage was observed in the tumor tissues of the other groups. Ki67 staining further confirmed that the “NTP-NO_2_ NPs + light” group had the lowest tumor cell proliferation. To investigate pyroptosis activation and the ICD effect of NPs, tumor tissues post-different treatments were analyzed using immunofluorescence and immunohistochemical imaging. As shown in Figure 6G-H, the “NTP-NO_2_ NPs + light” group exhibited decreased caspase-1 expression, increased GSDMD-N, and significantly triggered CRT exposure and HMGB1 efflux in tumor tissues. This observation *in vivo* is consistent with the pyroptosis and ICD effects observed at the cellular level. Immune activation was also assessed in each group by CD3⁺/CD8⁺ immunofluorescence staining. As shown in Figure 6I, significantly elevated CD3⁺/CD8⁺ expression in the “NTP-NO_2_ NPs + light” group indicated robust immune activation compared to other groups. These results indicate that the depletion of intracellular GSH by NTP-NO_2_ NPs can enhance PDT-induced pyroptosis and subsequently immune activation.

Mouse body weights were monitored every two days and steadily increased throughout the experiment (Figure S44). The absence of significant pathological alterations or tissue injury in the major organs (heart, liver, spleen, lungs, and kidneys) was demonstrated by H&E staining in all groups (Figure S45). Furthermore, heart, liver, and kidney functions were assessed by measuring serum biomarkers, including alanine aminotransferase (ALT), aspartate aminotransferase (AST), blood urea nitrogen (BUN), uric acid (UA), creatine kinase (CK), and LDH. As shown in the Figure S46, ALT, AST, BUN, UA, and CK were within normal range in all groups. Except for the “NTP-NO_2_ NPs + light” group, all other groups showed elevated LDH levels and failed to inhibit tumor growth; in contrast, the tumor-cured “NTP-NO_2_ NPs + light” group exhibited normalized CK levels. This indicates that PDT treatment with NTP-NO_2_ NPs can ameliorate liver and kidney impairment in tumor-bearing mice. To further evaluate the efficacy and safety of the different treatments, whole blood examinations were also performed to check (Table S2). Compared with non-tumor-bearing mice, erythrocyte and platelet counts in all experimental groups remained within the normal range. In contrast, leukocyte counts were elevated in the first four groups, whereas those in the tumor-eliminated “NTP-NO_2_ NPs + light” group returned to normal. These findings indicate that tumor implantation induces liver and kidney injury, along with immune and inflammatory responses, leading to elevated UA levels and leukocyte counts; treatment with “NTP-NO_2_ NPs + light” inhibited tumor growth and restored these physiological indicators to normal.

Since breast cancer often progresses to metastatic breast cancer 49, we employed a lung metastasis model to evaluate the antimetastatic capability of NTP-NO_2_ NPs against 4T1 cells following PDT. The model establishment scheme is illustrated in Figure S47A. After treatment, lung metastasis was assessed through Bouin's solution fixation (Figure S47B). The lung weight in the “NTP-NO_2_ NPs + light” group was significantly reduced compared with the other groups (Figure S47C). As shown in Figure S47D, no visible metastatic foci were observed in the lungs of mice treated with NTP-NO_2_ NPs under light irradiation, and the number of pulmonary nodules was significantly lower than that in the PBS control group. Moreover, consistent results were obtained from H&E staining of lung tissues (Figure S47E). In summary, these findings demonstrate that NTP-NO_2_ NPs exhibit excellent antimetastatic efficacy under PDT conditions.

## Conclusions

In summary, we report a chemically engineered strategy for controllable, tumor-selective pyroptosis by integrating GSH depletion and ROS restoration into AIE PS. Structure-function optimization of dono-acceptor architectures identified NTP-NO_2_, a π-extended acene derivative bearing a para-dinitrophenoxybenzyl pyridinium quencher, as a GSH-activated PS. This design enables selective activation in GSH-rich tumor cells, where it depletes intracellular antioxidants, triggers caspase-1/gasdermin-D-dependent pyroptosis, and amplifies ICD. Nanoparticle delivery of NTP-NO_2_ achieved high tumor accumulation, precise imaging, and pronounced antitumor efficacy *in vivo* without significant systemic toxicity. These findings demonstrate a molecular framework for developing PSs that couple chemical selectivity with immune-enhancing therapeutic mechanisms.

## Methods

### GSH depletion by MTP-NO_2_ NPs and NTP-NO_2_ NPs

After seeding 4T1 cells in 6-well plates and culturing them overnight, replaced the medium with a serum-free medium containing equal concentrations (60 µg/mL) of MTP NPs, NTP NPs, MTP-NO_2_ NPs, or NTP-NO_2_ NPs and incubated for 4 h. Subsequently, the light group was exposed to a 530 nm laser (100 mW/cm^2^, 10 min), while the dark group was not irradiated. After washing three times with PBS, the relative intracellular GSH level was determined according to the instructions of the GSH/GSSG Assay Kit.

### IL-1β, IL-18 and LDH release measurement

The concentrations of IL-1β and IL-18 in the supernatants were quantified with commercial enzyme-linked immunosorbent assay (ELISA) kits specific for each cytokine. LDH was measured using an LDH assay kit: 4T1 cells were seeded in 96-well plates at a density of 1 × 10^4^ cells per well and cultured overnight. The culture medium was then replaced with fresh medium containing 1% serum and nanoparticles (NTP NPs or NTP-NO_2_ NPs at 60 µg/mL). After 4 h of incubation, the light group was irradiated with a 530 nm laser (100 mW/cm^2^, 10 min), while the PBS group was kept in the dark as a control. All groups were further incubated for 6 h. Finally, the LDH activity in the supernatant was measured at 450 nm using a microplate reader. The percentage of LDH release was calculated as follows:

LDH release (%) = (LDH _treated_ - LDH _untreated cells_) / (LDH _total lysis_ - LDH _untreated cells_) × 100%

### *In vivo* fluorescence imaging

To assess the tumor-targeting efficiency of NTP NPs and NTP-NO_2_ NPs, their penetration and accumulation were analyzed in mice bearing 4T1 subcutaneous tumors. When the tumor volume reached approximately 200 mm^3^, the nanoparticles (6 mg/kg in 200 µL) were intravenously injected via the tail vein (n = 3). Whole-body fluorescence imaging was conducted at predetermined time points (0, 1, 2, 4, 8, 12, 24, 36, and 48 h post-injection) using a small animal imaging system with an excitation wavelength of 500 nm and a Cy5.5 emission filter. At 48 h post-injection, the mice were euthanized, and the tumors and major organs (heart, liver, spleen, lungs, and kidneys) were excised for *ex vivo* fluorescence imaging.

To validate the effect of tumor GSH on fluorescence response imaging of NTP-NO_2_ NPs, one group of tumor-bearing mice received an intratumoral injection of NEM (5 mM in 100 µL PBS, pH 7.4) 1 h before the NTP-NO_2_ NPs injection. In contrast, the two control groups were intravenously injected with NTP NPs or NTP-NO_2_ NPs (6 mg/kg, 200 µL) without NEM pretreatment. Tumor fluorescence was imaged 4 h post-injection using a small animal imaging system.

To observe fluorescence differences between normal and tumor tissues for NTP-NO_2_ NPs, tumors and surrounding tissues were collected at the time point of maximum nanoparticle accumulation after tail vein injection. Tissues underwent dehydration, paraffin embedding, and cryostat sectioning. Tissue sections were stained with DAPI and imaged by CLSM to examine nanoparticle distribution and surrounding tissue fluorescence intensity. H&E staining was simultaneously performed to distinguish the boundary between tumor tissue and normal tissue.

## Supplementary Material

Supplementary methods, figures and tables.

## Figures and Tables

**Table 1:** Summary of optical properties.

PSs	*λ*_ex_ (nm)	*λ*_em_ (nm)	*Φ*_F_ (%)^[a]^	^1^O_2_ production^[b]^	ROS production^[b]^	•OH production^[b]^
MTP	474	673	0.38	1	1	1
MTP-NO_2_	510	696	/	/	0.88	0.67
NTP	456	614	11.4	2.46	1.11	2.73
NTP-NO_2_	490	665	/	/	0.67	0.87

[a] DCM, with a known *Φ*_F_ of 43.5%, served as the reference for the measurement of the fluorescence quantum yields. [b] Relative ^1^O_2_, ROS or •OH production capacities by referencing MTP aggregate, which were all customized as 1.
