# Supplementary material for: Glutathione-depleting photosensitizers for tumor-specific imaging and pyroptosis-driven photodynamic therapy
Source: Theranostics. 2026 Jan 1;16(4):1818–32. doi: 10.7150/thno.124154 (PMC12680526; doi:10.7150/thno.124154)
Supplement: Supplementary file 1 — Supplementary methods, figures and tables. [file thnov16p1818s1.pdf]

Glutathione-depleting photosensitizers for tumor-specific imaging and pyroptosis-driven photodynamic therapy

Hui Yang,[1] Yifei Yang,[2] Zhengyumeng Zhu,[1] Pei Chen,[2] Jucai Gao,[1] Yajun Lin,[2] Qiang Wang,[2,*] and Fang Hu[1,3,*]

Experimental Procedures

Materials and instrumentation

4-hydroxy-benzaldehyde, 2,4-dinitrofluorobenzene, K_2CO_3, phosphorus tribromide, 4-bromo-N,N-bis-(4-methoxyphenyl)aniline, (5-formylthiophen-2-yl)boronic acid, [1,1'-bis (diphenylphosphino) ferrocene] palladium dichloride, sodium hydroxide, N-phenylnaphthalen-2-amine, 1-bromo-4-iodobenzene, t-BuONa, $Pd_2(dba)_3$ and $P(t\text{-}Bu)_3$ were provided by Adamas. DSPE-PEG2000 was purchased from Yuanye biotechnology. Glutathione was purchased from Macklin. 9,10-Anthracenediyl-bis(methylene)dimalonic acid (ABDA) was purchased from Sigma. Hydroxyphenyl fluorescein (HPF) was purchased from MKbio biotechnology. 2',7'-Dichlorofluorescein diacetate (DCFH-DA), 3-(4,5-dimethylthiazol-2-yl)-2,5-diphenyltetrazolium bromide (MTT), ATP Assay Kit, GSH and GSSG Assay Kit, Triton X-100 and Calcein-AM/PI Double Stain Kit were provided by beyotime. HMGB1 antibody (3935S), CRT Antibody (ab92516), Gasdermin D (E9S1X) Rabbit mAab (39754) and Caspase-1 (E2Z1C) Rabbit mAb (24232) were purchased from CST. All commercial chemicals were used as received without further purification. Compounds 4-(2,4-dinitrophenoxy)benzaldehyde, (4-(2,4-dinitrophenoxy)phenyl)methanol, 1-(4-(bromomethyl)phenoxy)-2,4-dinitrobenzene were synthesized according to the reported literature.

^{1}H NMR and ^{13}C NMR spectra were recorded on a Bruker AVANCE III 400 MHz spectrometer using tetramethylsilane (TMS; δ = 0 ppm) as an internal standard. Electrospray ionization mass spectrometry (ESI-MS) analyses were performed on an LC-MS system (Thermo Scientific, USA).. Matrix Assisted Laser Desorption Ionization (MALDI) mass

spectra were acquired on a Bruker Daltonics UltrafleXtreme time of flight (TOF) equipment. Hydrodynamic diameter and size distribution were measured on a NanoBrook 90PlusZeta (Brookhaven Instruments Corporation) and morphological observations were conducted by transmission electron microscopy (TEM) on a JEOL-1400 PLUS instrument.. Cytotoxicity tests were carried out on a microplate reader (Envision, PerkinElmer) by using MTT assays. Confocal Laser Scanning Microscopy (CLSM) images were captured by NIS-Elements 5.3 (Nikon). The light power intensity was measured by LWP10W-A optical power meter (Beijing Laserwave OptoElectronics Technology Co., Ltd). All PDT experiments were performed using an LWGL532-2W laser source.

Synthetic procedure

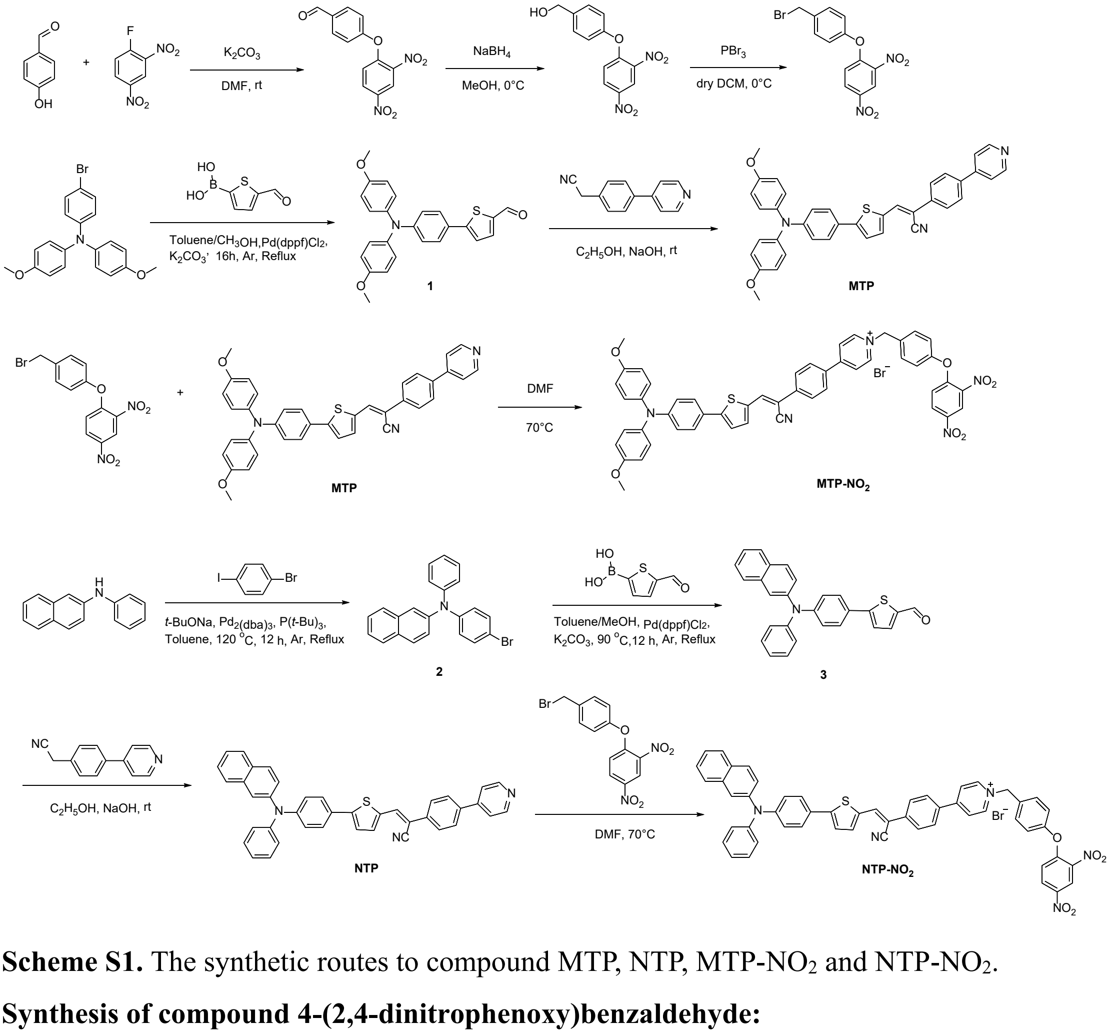

Scheme S1. The synthetic routes to compound MTP, NTP, MTP-NO_2 and NTP-NO_2.

Synthesis of compound 4-(2,4-dinitrophenoxy)benzaldehyde:

To a solution of 4-hydroxy-benzaldehyde (1500.0 mg, 12.28 mmol) and K_2CO_3 (3400.0 mg, 24.60 mmol) in DMF (15 mL), 2,4-dinitrofluorobenzene (2500.0 mg, 13.43 mmol) was added

and then the reaction mixture was stirred for 14 h under argon atmosphere at room temperature. After cooling to room temperature, the reaction mixture was extracted with dichloromethane and H_2O. The organic layer was dried over Na_2SO_4 and then concentrated under vacuum. The residue was purified by column chromatography on silica gel with petroleum ether/ethyl acetate (5:1, v/v) as the eluent to afford the compound 4-(2,4-dinitrophenoxy)benzaldehyde as a yellow solid. Yield: 82%. ^{1}H NMR (400 MHz, Chloroform-*d*) δ 10.03 (s, 1H), 8.90 (d, J = 2.7 Hz, 1H), 8.43 (dd, J = 9.2, 2.8 Hz, 1H), 8.05 – 7.97 (m, 2H), 7.28 (d, J = 2.0 Hz, 1H), 7.26 (d, J = 2.0 Hz, 1H), 7.20 (d, J = 9.1 Hz, 1H).

Synthesis of compound (4-(2,4-dinitrophenoxy)phenyl)methanol:

To a solution of compound 4-(2,4-dinitrophenoxy)benzaldehyde (2000.0 mg, 6.94 mmol) in MeOH, sodium borohydride (262.0 mg, 6.93 mmol) was added and then the reaction mixture was stirred for 6 h under argon atmosphere at 0 °C. The reaction was quenched with deionized water and extracted with CH_2Cl_2 and H_2O. The organic layer was dried over Na_2SO_4, filtered, and then concentrated under vacuum. The residue was purified by column chromatography on silica gel with petroleum ether/ethyl acetate (2:1, v/v) as the eluent to afford the compound (4-(2,4-dinitrophenoxy)phenyl)methanol as a yellow solid. Yield: 57%. ^{1}H NMR (400 MHz, Chloroform-*d*) δ 8.86 (d, J = 2.8 Hz, 1H), 8.32 (dd, J = 9.3, 2.8 Hz, 1H), 7.50 (d, J = 8.4 Hz, 2H), 7.19 – 7.10 (m, 2H), 7.03 (d, J = 9.3 Hz, 1H), 4.76 (d, J = 4.8 Hz, 2H).

Synthesis of compound 1-(4-(bromomethyl)phenoxy)-2,4-dinitrobenzene:

To a solution of compound (4-(2,4-dinitrophenoxy)phenyl)methanol (440.0 mg, 1.52 mmol) in dry dichloromethane, phosphorus tribromide (0.55 mL) was added slowly and then the reaction mixture was stirred for 6 h under argon atmosphere at 0 °C. After cooling to room temperature, the reaction mixture was quenched with sodium bicarbonate saturated solution and extracted with ethyl acetate and H_2O. The organic layer was dried over Na_2SO_4, filtered, and then concentrated under vacuum. The residue was purified by column chromatography on silica gel with petroleum ether/ethyl acetate (4:1, v/v) as the eluent to afford compound 1-(4-(bromomethyl)phenoxy)-2,4-dinitrobenzene as a white solid. Yield: 62%. ^{1}H NMR (400 MHz, Chloroform-*d*) δ 8.86 (d, J = 2.8 Hz, 1H), 8.34 (dd, J = 9.2, 2.8 Hz, 1H), 7.60 – 7.45 (m, 2H), 7.17 – 7.10 (m, 2H), 7.08 (d, J = 9.3 Hz, 1H), 4.53 (s, 2H).

Synthesis of compound 1:

4-bromo-*N,N*-bis-(4-methoxyphenyl)aniline (1500.0 mg, 3.90 mmol), (5-formylthiophen-2-yl)boronic acid (792.0 mg, 5.08 mmol), [1,1'-bis (diphenylphosphino) ferrocene] palladium dichloride (428.4 mg, 0.59 mmol) and potassium carbonate (2700.0 mg, 19.54 mmol) were added to a 250 mL round-bottom flask. Subsequently, 20 mL mixture of toluene and methanol was added at a volume ratio of 1:1. Then the reaction mixture was stirred and refluxed at 80 ºC for 16 h under argon atmosphere. After cooling to room temperature, the reaction solvent was evaporated under reduced pressure. And the reaction mixture was extracted with dichloromethane and H_2O. The organic layer was dried over Na_2SO_4, filtered, and then concentrated under vacuum. The residue was purified by column chromatography on silica gel with petroleum ether/ethyl acetate (15:1, v/v) as the eluent to afford the compound **1** as a red solid. Yield: 95%.^{1}H NMR (400 MHz, Chloroform-*d*) δ 9.84 (d, J = 2.6 Hz, 1H), 7.69 (dd, J = 4.1, 2.6 Hz, 1H), 7.46 (dd, J = 9.0, 2.5 Hz, 2H), 7.27 (s, 1H), 7.10 (dd, J = 9.0, 2.5 Hz, 4H), 6.96 – 6.81 (m, 6H), 3.81 (d, J = 2.6 Hz, 6H).

Synthesis of compound MTP:

To a solution of compound **1** (140.0 mg, 0.34 mmol) and 2-(4-pyridin-4-ylphenyl)acetonitrile (70.0 mg, 0.36 mmol) in ethanol, sodium hydroxide (17.0 mg, 0.43 mmol) was added and then the reaction mixture was stirred for 5 h at room temperature. The solid was filtered and extracted with dichloromethane and H_2O to remove NaOH. The organic layer was dried over Na_2SO_4, filtered, and then concentrated under vacuum. The solid was recrystallized with ethyl acetate and petroleum ether to afford the compound **MTP** as a red solid. Yield: 81%. ^{1}H NMR (400 MHz, Chloroform-*d*) δ 8.70 (t, J = 5.9 Hz, 2H), 7.76 (dd, J = 8.4, 4.9 Hz, 2H), 7.73 – 7.68 (m, 2H), 7.62 (d, J = 8.3 Hz, 1H), 7.60 – 7.56 (m, 1H), 7.56 – 7.53 (m, 1H), 7.50 – 7.45 (m, 2H), 7.24 – 7.15 (m, 2H), 7.12 – 7.07 (m, 2H), 7.06 – 7.01 (m, 2H), 6.94 – 6.90 (m, 1H), 6.88 – 6.85 (m, 2H), 6.81 (dd, J = 8.9, 6.8 Hz, 3H), 3.80 (d, J = 12.8 Hz, 6H). ^{13}C NMR (101 MHz, Chloroform-*d*) δ 156.38, 150.44, 149.45, 147.15, 140.10, 138.05, 135.48, 135.05, 134.84, 127.61, 127.08, 126.93, 126.16, 124.74, 122.14, 121.58, 121.38, 119.66, 119.40, 118.37, 114.84, 105.14, 55.53.

Synthesis of compound MTP-NO_2:

Compound 1-(4-(bromomethyl)phenoxy)-2,4-dinitrobenzene (40.0 mg, 0.11 mmol) and compound **MTP** (20.0 mg, 0.03 mmol) were dissolved in dimethylformamide (3 mL) and then

the mixture was stirred at 70 °C overnight. After cooling to room temperature, the product was precipitated *via* the addition of 50 mL of diethyl ether and filtered to afford the compound **MTP-NO_2** as a black solid. Yield: 94%. ^{1}H NMR (400 MHz, DMSO-d_6) δ 9.29 (s, 2H), 8.92 (s, 1H), 8.64 (s, 2H), 8.50 (d, J = 15.5 Hz, 2H), 8.24 (s, 2H), 7.98 (s, 2H), 7.79 (s, 3H), 7.59 (s, 3H), 7.39 (s, 2H), 7.23 (s, 1H), 7.11 (s, 4H), 6.96 (s, 4H), 6.80 (s, 2H), 5.89 (s, 2H), 3.76 (s, 6H). ^{13}C NMR (101 MHz, DMSO-d_6) δ 156.77, 155.07, 154.73, 154.33, 150.69, 149.74, 145.27, 142.26, 140.18, 139.67, 138.63, 137.82, 135.41, 133.30, 132.58, 131.98, 130.13, 129.54, 127.80, 127.44, 126.59, 125.16, 124.10, 123.37, 122.42, 121.17, 120.42, 118.85, 118.45, 115.55, 103.32, 61.98, 55.74.

Synthesis of compound 2:

To a solution of *N*-phenylnaphthalen-2-amine (1100.0 mg, 5.02 mmol) and 1-bromo-4-iodobenzene (1560.0 mg, 5.52 mmol) and *t*-BuONa (1400.0 mg, 15.06 mmol) in toluene (25 mL), $Pd_2(dba)_3$ (91 mg, 0.099 mmol) and P(*t*-Bu)$_3$ (20.0 mg, 0.099 mmol) was added and then the reaction mixture was stirred and refluxed for 12 h under argon atmosphere at 120 °C. After cooling to room temperature, the reaction solvent was evaporated under reduced pressure. The reaction mixture was extracted with dichloromethane and H_2O. The organic phase was concentrated under reduced pressure. The residue was purified by column chromatography on silica gel with petroleum ether/dichloromethane (50:1, v/v) as the eluent to afford compound **2** as a white oily solid. Yield: 46.0%. ^{1}H NMR (400 MHz, Chloroform-*d*) δ 7.74 (dd, J = 13.0, 8.3 Hz, 2H), 7.59 (d, J = 7.9 Hz, 1H), 7.45 – 7.31 (m, 5H), 7.31 – 7.21 (m, 3H), 7.12 (d, J = 8.0 Hz, 2H), 7.06 (t, J = 7.3 Hz, 1H), 7.03 – 6.95 (m, 2H).

Synthesis of compound 3:

To a solution of compound **2** (215.6 mg, 0.58 mmol) and (5-formylthiophen-2-yl)boronic acid (192.0 mg, 1.23 mmol) in toluene and methanol solution (26 mL), K_2CO_3 (690 mg, 5 mmol) and Pd(dppf)Cl_2 (59.0 mg, 0.081 mmol) was added and then the reaction mixture was stirred and refluxed for 18 h under argon atmosphere at 90 °C. After cooling to room temperature, the reaction solvent was evaporated under reduced pressure. The reaction mixture was extracted with dichloromethane and H_2O. The organic phase was concentrated under reduced pressure. The residue was purified by column chromatography on silica gel with petroleum ether/dichloromethane (3:1, v/v) as the eluent to give the compound **3** as a yellowish oily solid.

Yield: 70.3%. ^{1}H NMR (400 MHz, Chloroform-*d*) δ 9.86 (d, J = 1.3 Hz, 1H), 7.78 (t, J = 7.7 Hz, 2H), 7.72 (t, J = 2.5 Hz, 1H), 7.63 (d, J = 7.7 Hz, 1H), 7.57 – 7.49 (m, 3H), 7.41 (m, J = 6.8 Hz, 2H), 7.35 – 7.27 (m, 4H), 7.19 (d, J = 7.9 Hz, 2H), 7.15 – 7.08 (m, 3H).

Synthesis of compound NTP:

To a solution of compound **3** (140.0 mg, 0.35 mmol) and 2-(4-pyridin-4-ylphenyl)acetonitrile (68.0 mg, 0.35 mmol) in ethanol, sodium hydroxide (17.0 mg, 0.43 mmol) was added and then the reaction mixture was stirred for 5 h at room temperature. A large amount of solid was produced from the reaction system. The solid was filtered and extracted with dichloromethane and H_2O to remove NaOH. The soild was recrystallized with trichloromethane and diethyl ether to afford the compound **NTP** as a red solid. Yield: 80%. ^{1}H NMR (600 MHz, Chloroform-*d*) δ 8.70 (s, 2H), 7.76 (t, J = 8.8 Hz, 3H), 7.74 – 7.68 (m, 2H), 7.62 (d, J = 8.4 Hz, 2H), 7.60 – 7.52 (m, 3H), 7.49 (d, J = 18.7 Hz, 1H), 7.46 – 7.36 (m, 3H), 7.29 (q, J = 8.8, 8.3 Hz, 4H), 7.26 – 7.16 (m, 2H), 7.14 – 7.06 (m, 4H), 7.02 (d, J = 8.7 Hz, 1H). ^{13}C NMR (151 MHz, Chloroform-*d*) δ 150.37, 150.32, 149.90, 149.86, 148.49, 148.42, 147.32, 147.23, 147.11, 147.01, 144.72, 144.61, 139.39, 138.16, 137.74, 136.07, 135.77, 134.99, 134.85, 134.79, 134.72, 134.38, 134.32, 133.44, 130.45, 130.43, 130.18, 129.52, 129.49, 129.19, 129.18, 127.96, 127.64, 127.62, 127.11, 127.07, 127.00, 126.87, 126.71, 126.46, 126.45, 126.23, 125.12, 125.09, 124.99, 124.94, 124.68, 124.63, 123.88, 123.85, 123.14, 122.91, 122.79, 122.08, 121.58, 121.48, 121.43, 120.35, 118.23, 107.78, 105.74.

Synthesis of compound NTP-NO_2:

Compound 1-(4-(bromomethyl)phenoxy)-2,4-dinitrobenzene (87.0 mg, 25 mmol) and compound **NTP** (72.0 mg, 0.12 mmol) were dissolved in dimethylformamide (10 mL) and then the mixture was stirred at 70 °C overnight. After cooling to room temperature, the product was precipitated *via* the addition of 50 mL of diethyl ether and filtered to afford the compound **NTP-NO_2** as a black solid. Yield: 56.6%. ^{1}H NMR (400 MHz, DMSO-d_6) δ 9.28 (d, J = 6.5 Hz, 2H), 8.92 (d, J = 2.8 Hz, 1H), 8.63 (d, J = 6.6 Hz, 2H), 8.53 (s, 1H), 8.47 (dd, J = 9.3, 2.8 Hz, 1H), 8.24 (d, J = 8.6 Hz, 2H), 7.98 (d, J = 8.6 Hz, 2H), 7.91 (d, J = 8.9 Hz, 1H), 7.87 (s, 1H), 7.83 (d, J = 4.1 Hz, 1H), 7.77 (d, J = 8.6 Hz, 3H), 7.71 (d, J = 8.7 Hz, 2H), 7.63 (s, 1H), 7.55 (d, J = 2.2 Hz, 1H), 7.49 – 7.42 (m, 2H), 7.42 – 7.34 (m, 4H), 7.28 (dd, J = 8.8, 2.3 Hz, 1H), 7.23 (d, J = 9.3 Hz, 1H), 7.20 – 7.12 (m, 3H), 7.09 (d, J = 8.8 Hz, 2H), 5.88 (s, 2H). ^{13}C NMR (101

MHz, DMSO-d_6) δ 155.10, 154.72, 154.34, 149.97, 148.56, 146.97, 145.29, 144.64, 142.28, 140.20, 138.52, 137.83, 137.70, 136.11, 134.47, 133.47, 132.58, 131.96, 130.56, 130.31, 130.13, 129.89, 129.57, 128.02, 127.68, 127.53, 127.05, 126.72, 125.59, 125.43, 125.22, 124.92, 124.63, 124.21, 123.08, 122.44, 121.56, 121.19, 120.41, 118.38, 103.96, 62.07.

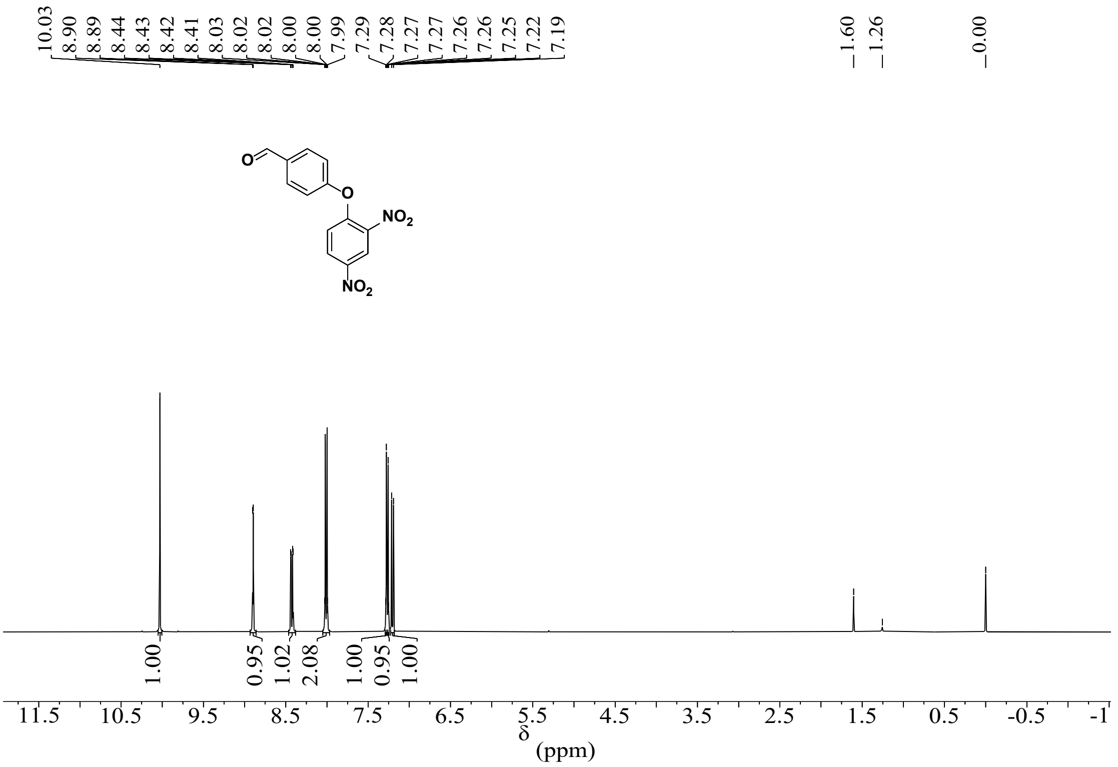

Figure S1. ^{1}H NMR spectrum of compound 4-(2,4-dinitrophenoxy)benzaldehyde in $CDCl_3$.

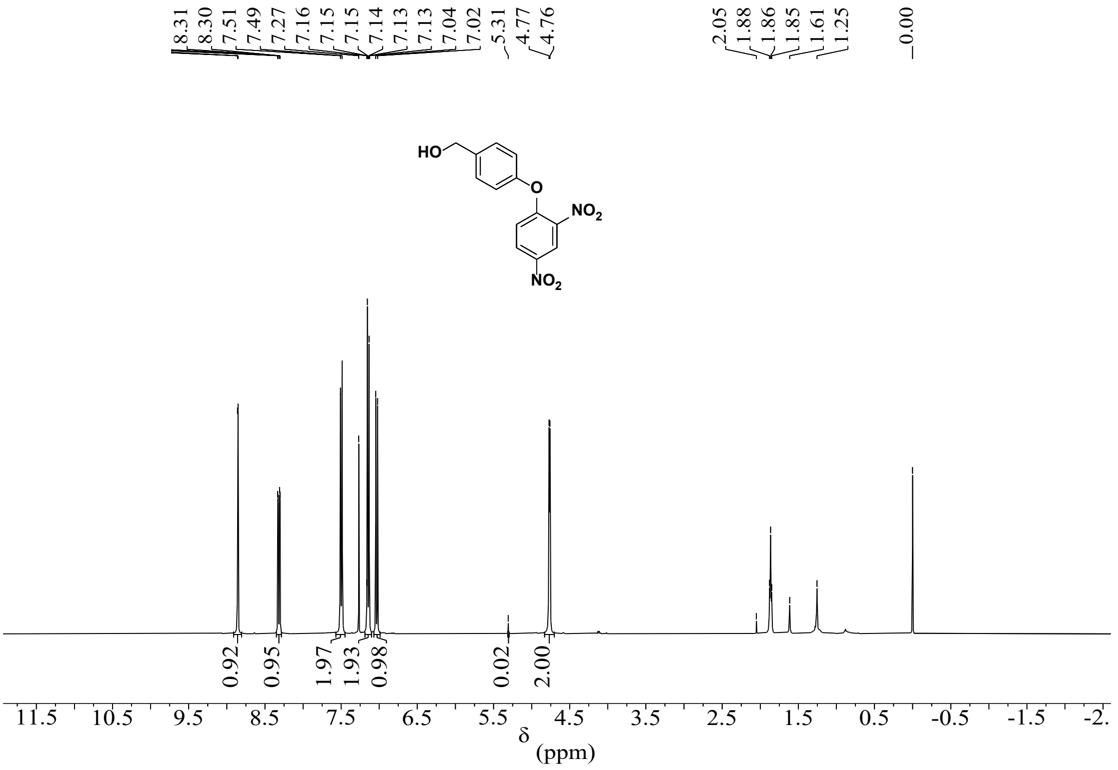

Figure S2. ^{1}H NMR spectrum of compound (4-(2,4-dinitrophenoxy)phenyl)methanol in $CDCl_3$.

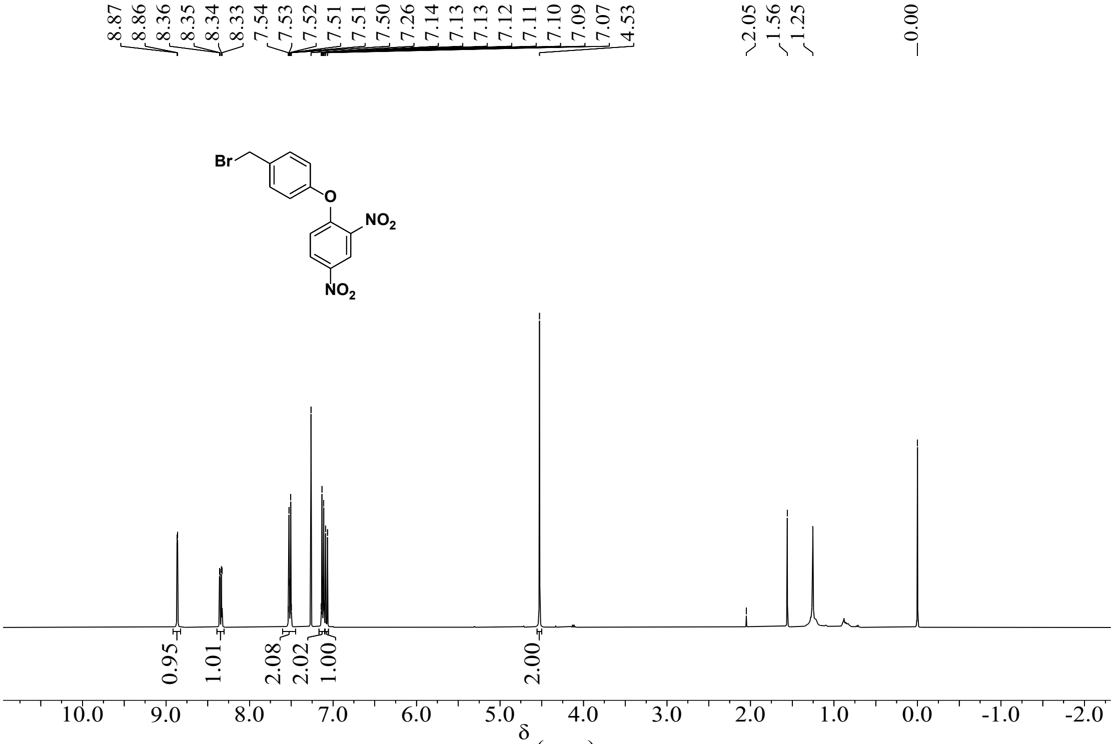

Figure S3. ^{1}H NMR spectrum of compound 1-(4-(bromomethyl)phenoxy)-2,4-dinitrobenzene in $CDCl_3$.

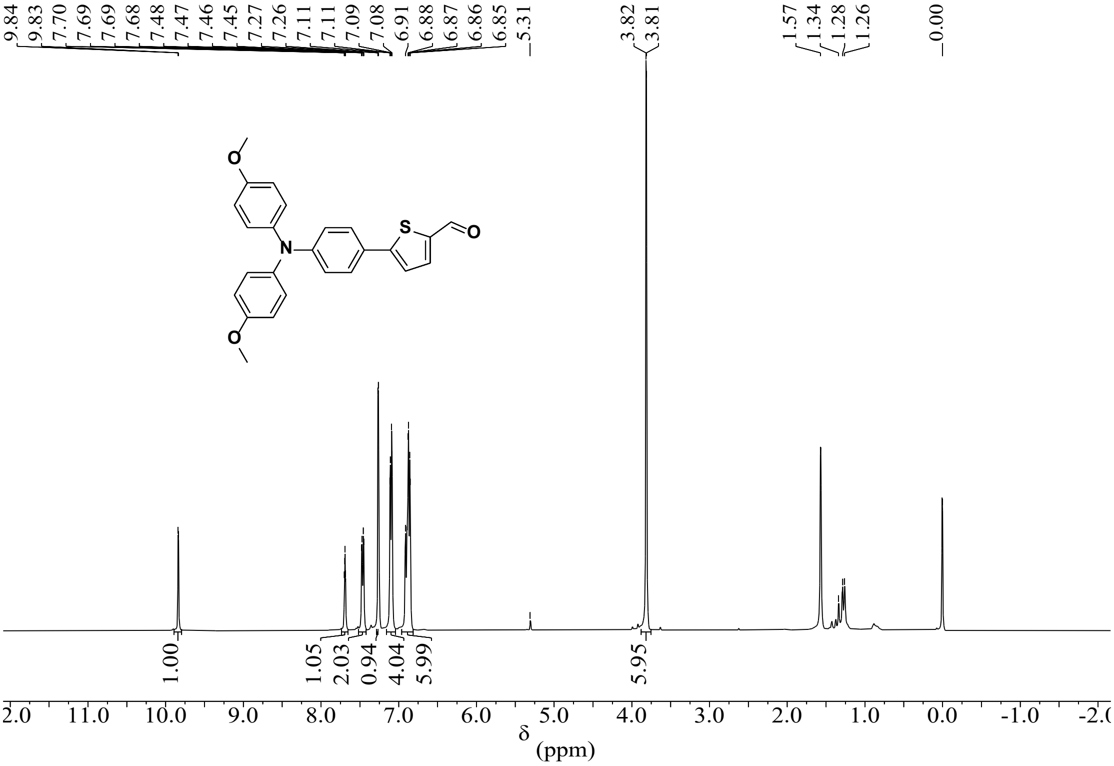

Figure S4. ^{1}H NMR spectrum of **compound 1** in $CDCl_3$.

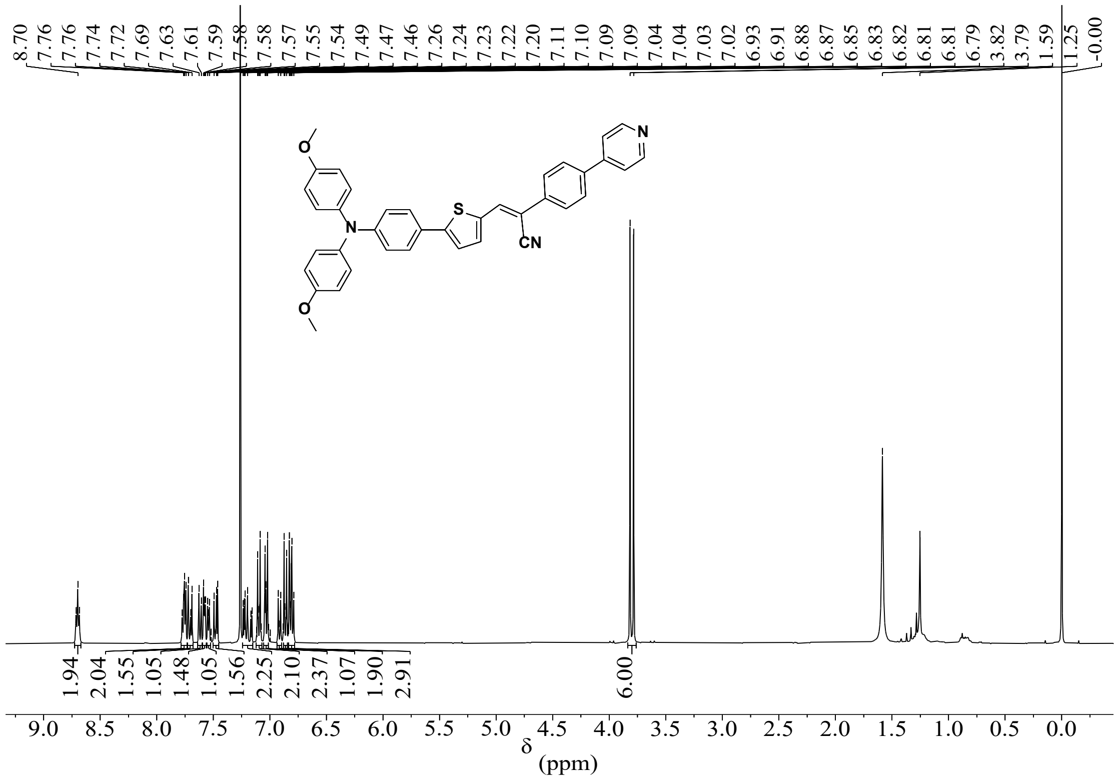

Figure S5. ^{1}H NMR spectrum of **compound MTP** in $CDCl_3$.

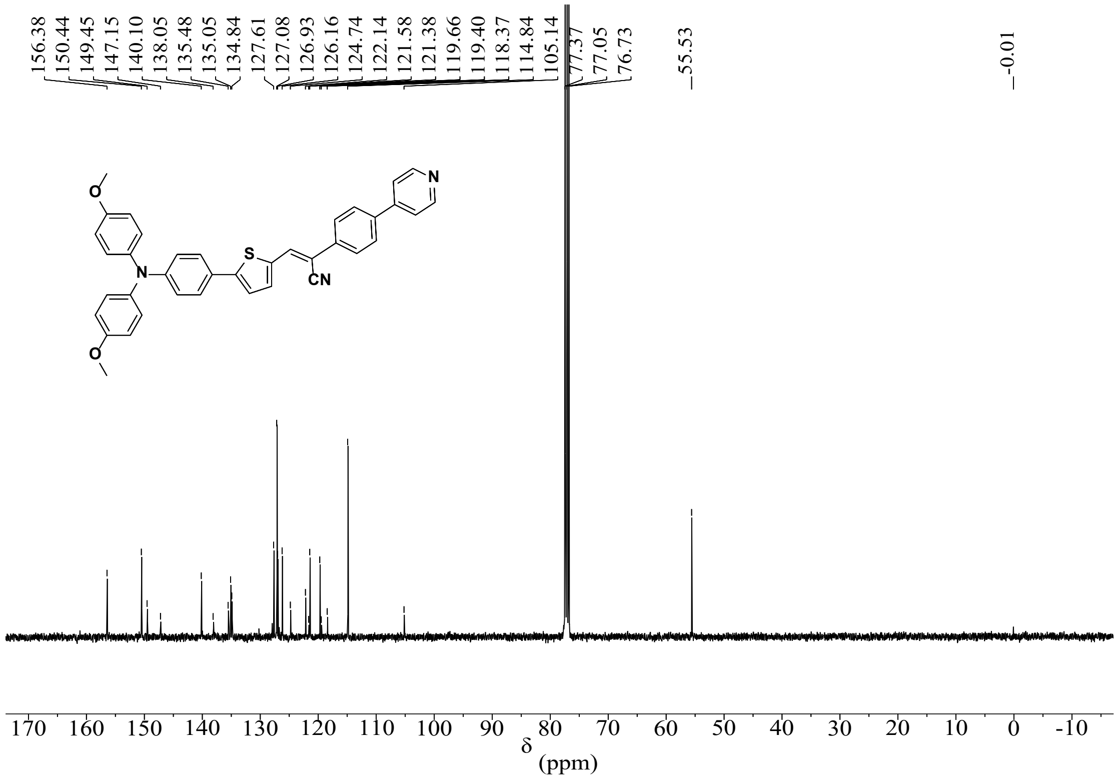

Figure S6. ^{13}C NMR spectrum of **compound MTP** in $CDCl_3$.

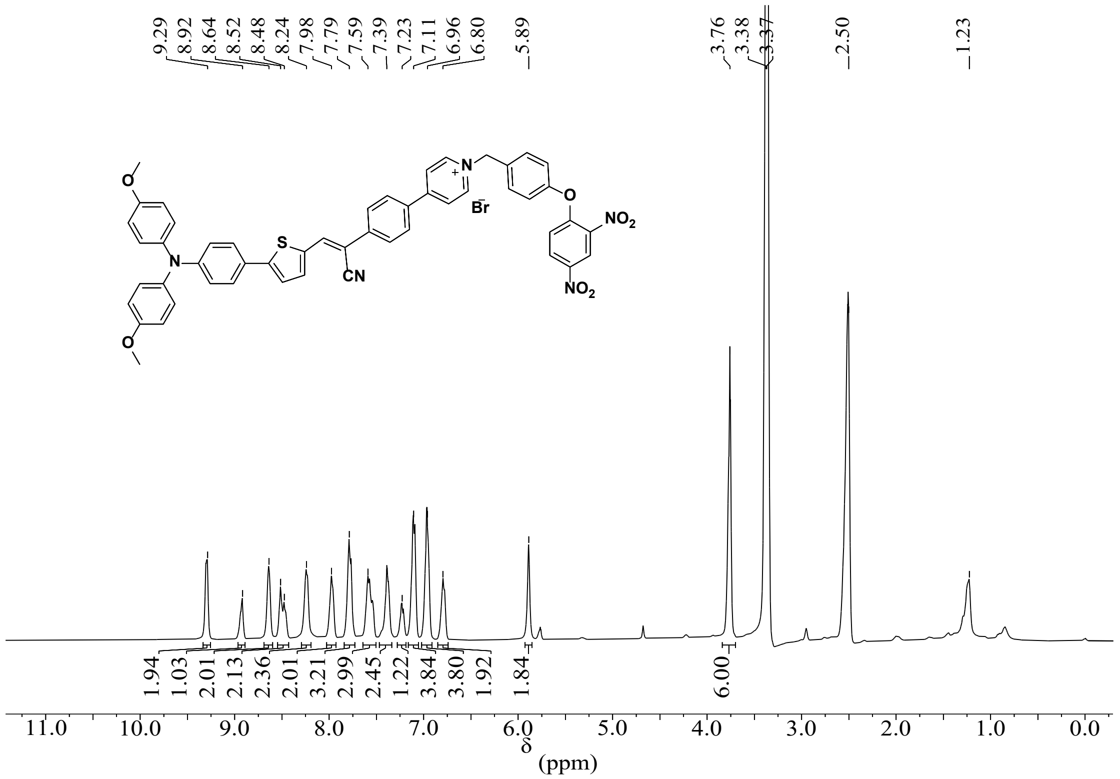

Figure S7. ^{1}H NMR spectrum of **compound MTP-NO₂** in DMSO-d_6.

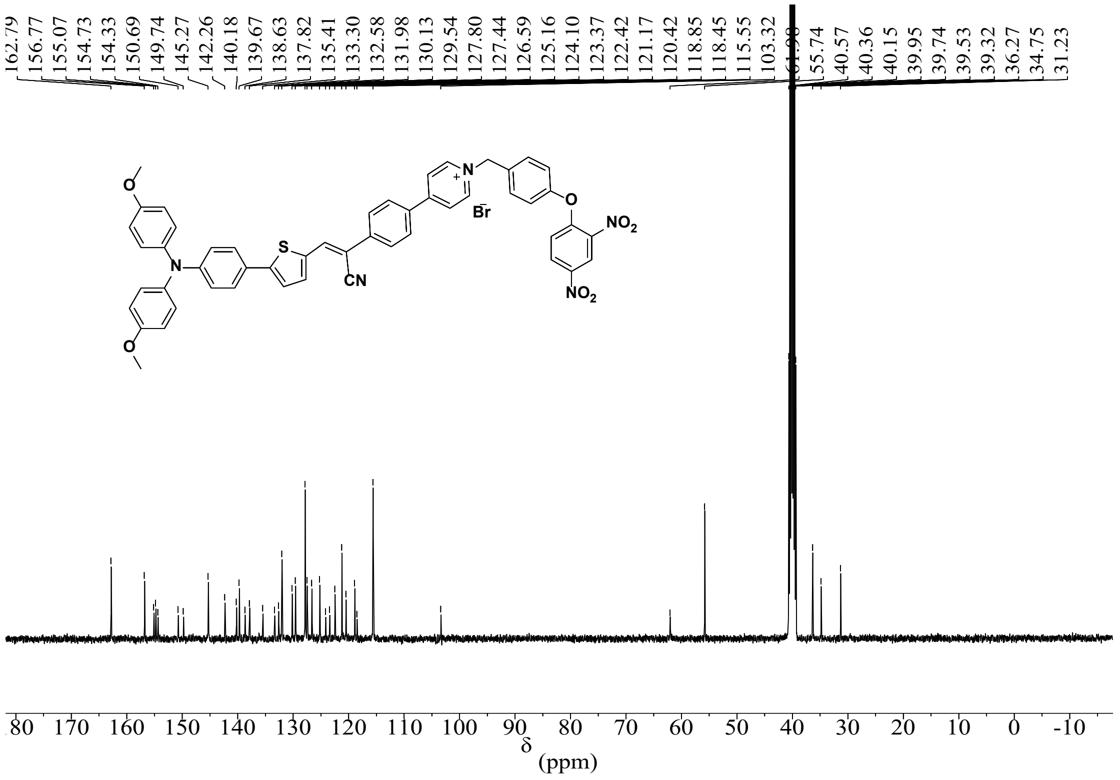

Figure S8. ^{13}C NMR spectrum of **compound MTP-NO₂** in DMSO-d_6.

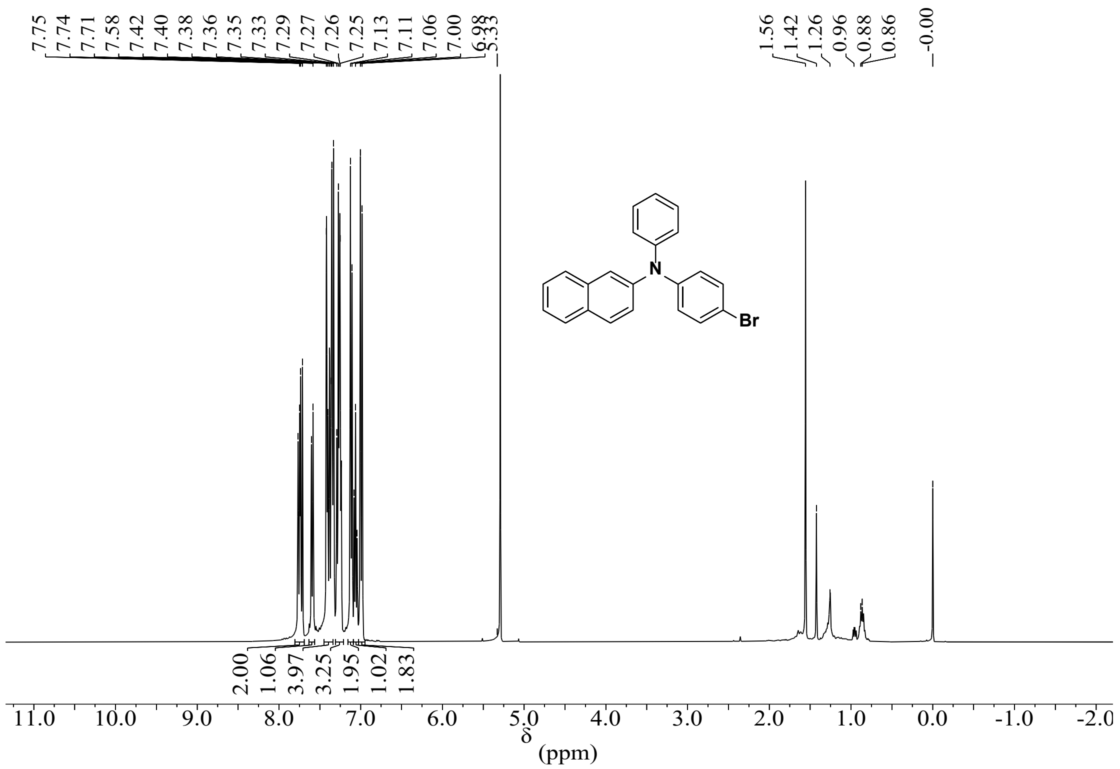

Figure S9. ^{1}H NMR spectrum of **compound 2** in $CDCl_3$.

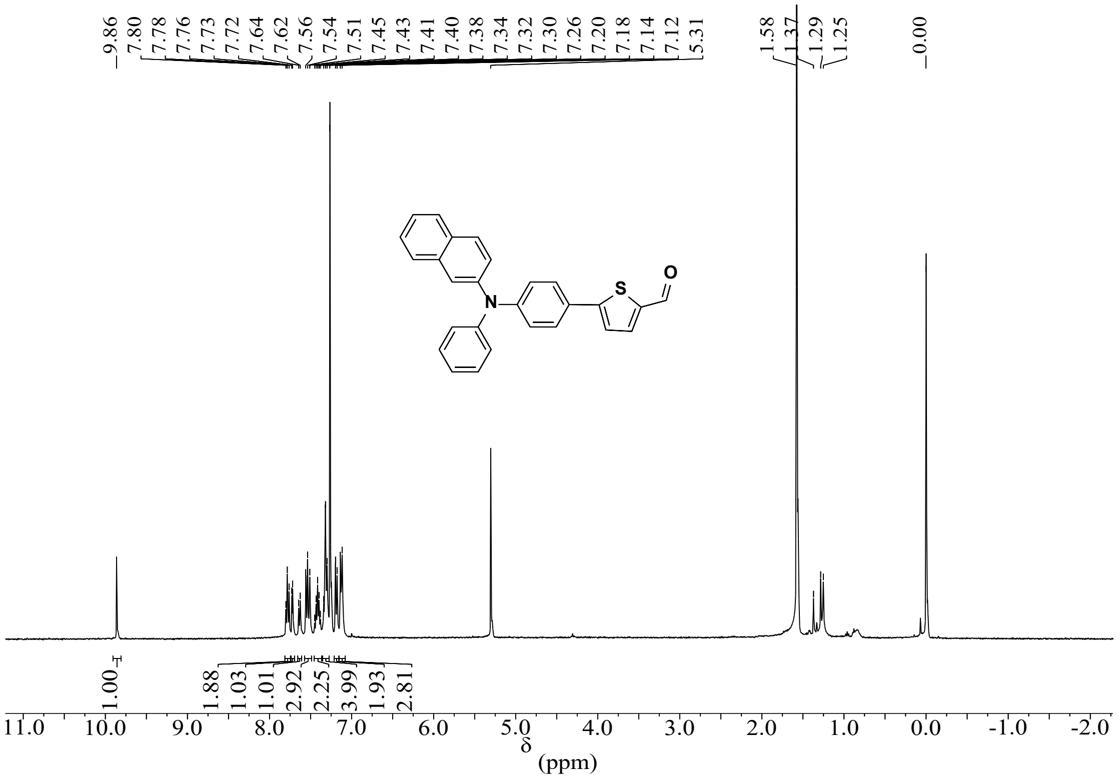

Figure S10. ^{1}H NMR spectrum of **compound 3** in $CDCl_3$.

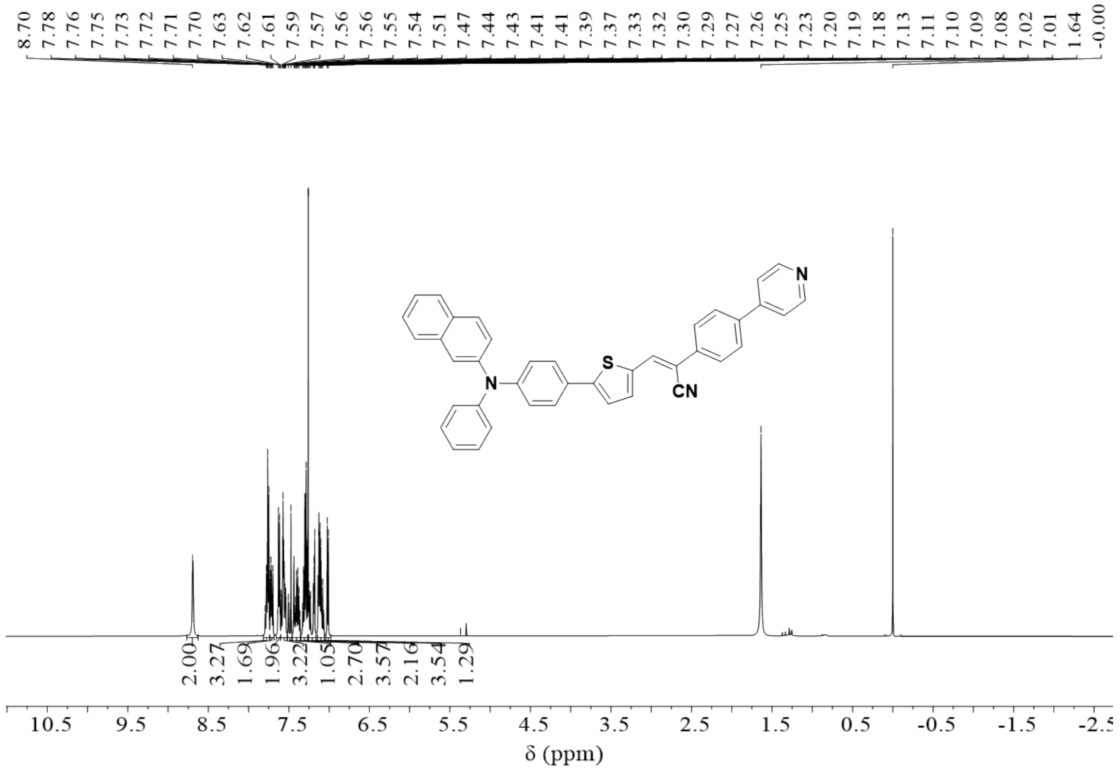

Figure S11. ^{1}H NMR spectrum of **compound NTP** in $CDCl_3$.

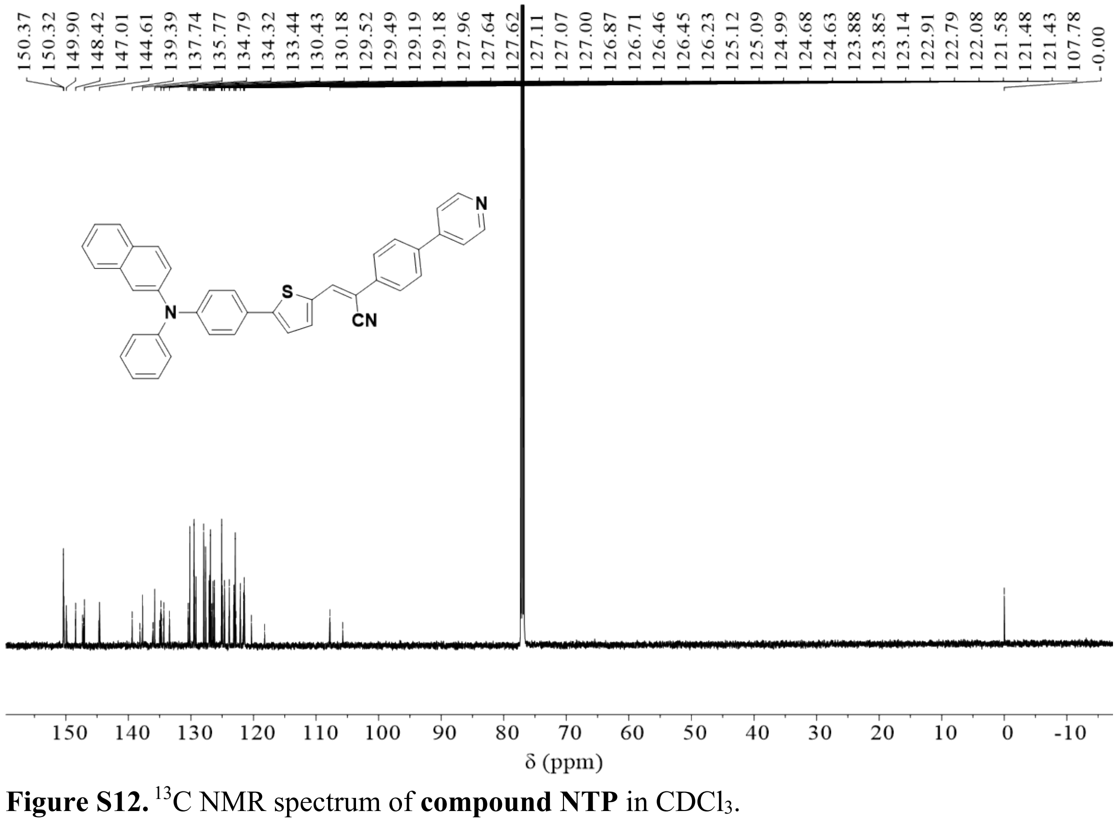

Figure S12. ^{13}C NMR spectrum of **compound NTP** in $CDCl_3$.

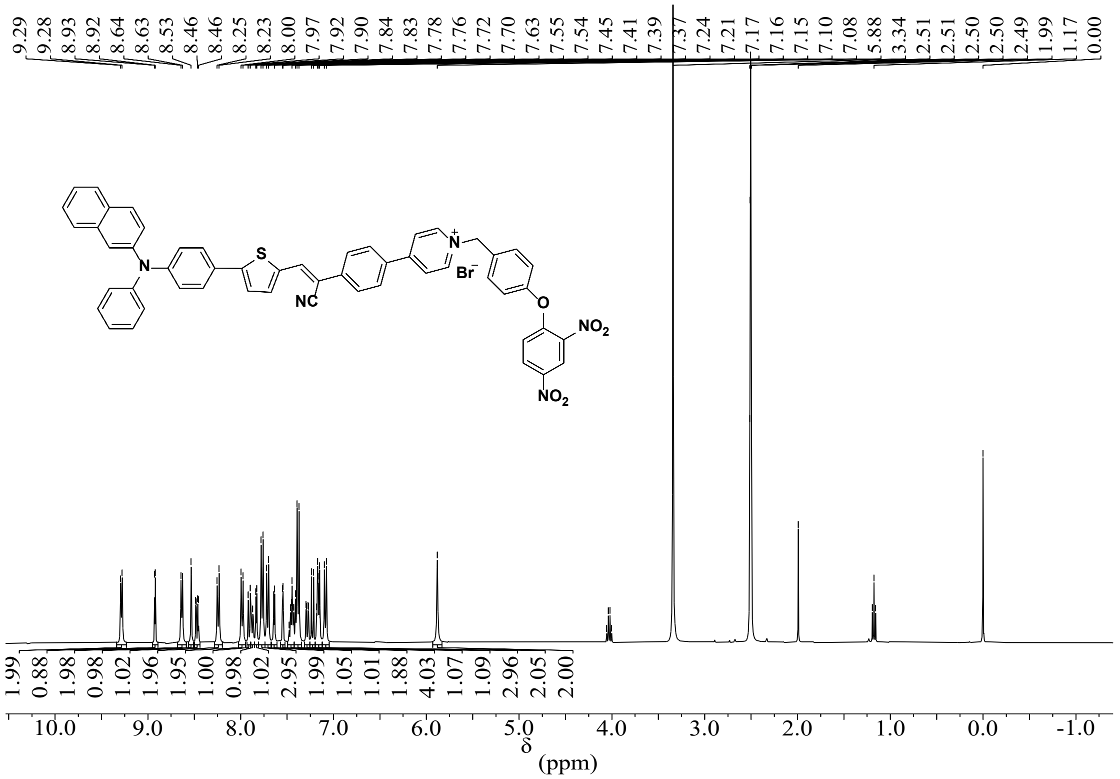

Figure S13. ^{1}H NMR spectrum of **compound NTP-NO$_2$** in DMSO-d_6.

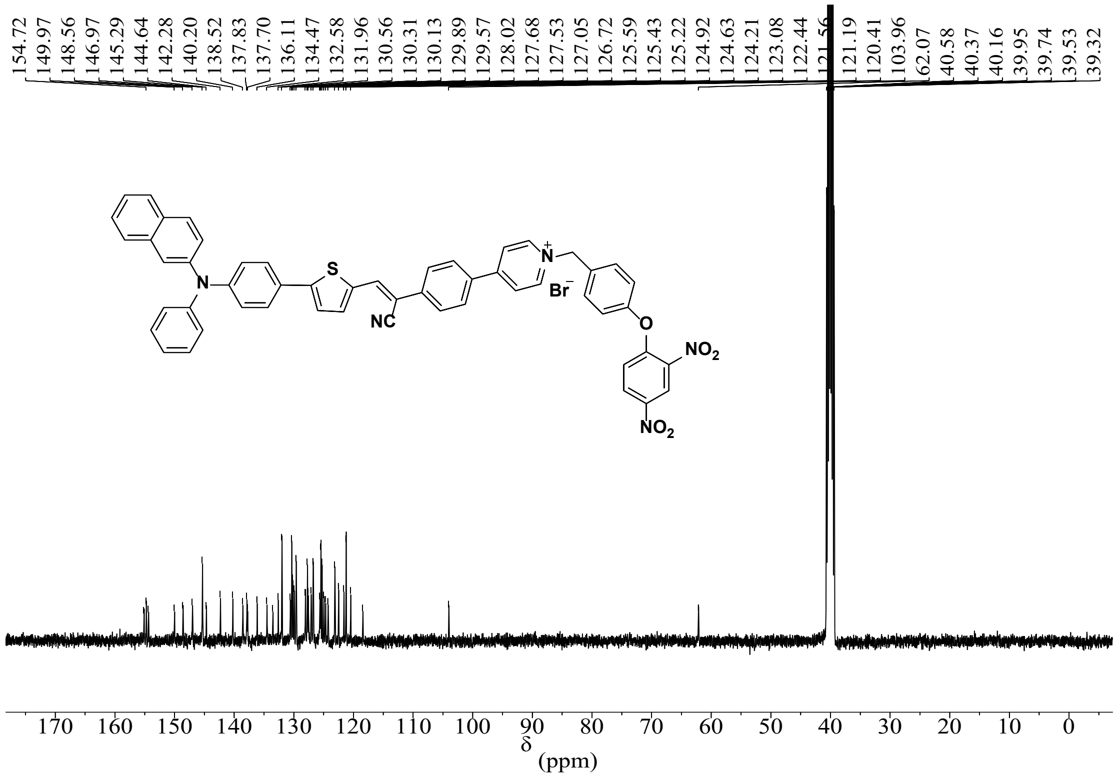

Figure S14. ^{13}C NMR spectrum of **compound NTP-NO$_2$** in DMSO-d_6.

Measurement of fluorescence quantum yield

The fluorescence quantum yield (Φ_F) of the MTP, NTP in aqueous media were measured using a standard fluorescence comparison method. Freshly prepared 4-(dicyanomethylene)-2-methyl-6-(4-dimethylaminostyryl)-4*H*-pyran (DCM) in methanol was used as reference (Φ_F = 43.5%). The UV-*vis* absorption spectra of compounds in aqueous media and DCM in methanol were measured and the maximum absorption was limited to less than 0.1 to minimize the self-absorption effect. The fluorescence spectra then were recorded under maximum excitation. The fluorescence intensities were obtained through wavelength integration. The Φ_F of AIE PSs was calculated by the following equation:

$$\Phi_x = \Phi_{st}\left(\frac{k_x}{k_{st}}\right)\left(\frac{\eta_x^2}{\eta_{st}^2}\right)$$

where Φ_{st} is the standard fluorescence quantum yield (Φ_{DCM} = 43.5%). k_x and k_{st} are is the slope after linear fitting of fluorescence intensity integral *vs.* the absorption of AIE PSs and DCM, respectively. η_x and η_{st} are the refractive indexes of the solvent. The subscripts x and st denote the sample and the reference (DCM in methanol).

Preparation of NPs

The mixed THF solution (1 mL) containing MTP, NTP, MTP-NO_2 or NTP-NO_2 (1 mg) and DSPE-PEG2000 (4 mg) was rapidly injected into water (10 mL) under sonication. After sonication for 3 min, THF was evaporated by stirring at room temperature overnight. The obtained NPs were filtered by a syringe-driven filter (0.22 μm) and stored at 4 °C in the dark.

ROS generating ability test

$^1O_2$: ABDA (100 μM) was added as a singlet oxygen indicator to the sample solution containing NPs. After irradiation with a 530 nm laser (100 mW/cm^2) for varying durations, the absorption spectra of ABDA were measured.

•OH: HPF (5×10^{-6} M) was added as a hydroxyl radical indicator to an aqueous sample solution containing NPs. The fluorescence spectra of the mixed solution after irradiation with a 530 nm laser (100 mW/cm^2) for varying durations were measured using a fluorescence spectrophotometer at 490 nm excitation.

ROS: Non-fluorescent H2DCF (2×10^{-5} M) reacts with ROS to form highly fluorescent DCF. H2DCF was added to an aqueous solution containing NPs. Changes in the fluorescence spectra

of the mixed solution after 530 nm laser irradiation (100 mW/cm^2) for varying durations were recorded using a fluorescence spectrophotometer.

Cell cultures

4T1 cells, HUVECs, MDA-MB-231 cells, MCF7 cells, and MCF10A were cultured in DMEM (containing 10% heat-inactivated fetal bovine serum and 1% Penicillin streptomycin) at 37 °C in a humidified incubator with 5% CO_2. Before the experiments, the cells were pre-cultured until confluence was reached.

GSH-induced fluorescence changes of MTP-NO_2 and NTP-NO_2 NPs

The incubation of nanoparticles with GSH was conducted in a mixed solution of PBS:DMSO = 3:1 (v/v). First, fluorescence changes over time were measured using a fluorescence spectrophotometer after incubating nanoparticles with GSH for 0, 10, 20, 30, 40, 50, 60, and 90 min (corresponding to GSH concentrations of 0.01, 0.05, 0.2, 0.5 mM). For fluorescence change tests of MTP-NO_2 NPs or NTP-NO_2 NPs incubated with varying GSH concentrations: probes were added to a mixed solution of PBS:DMSO = 3:1 (v/v) along with different GSH concentrations (0, 0.01, 0.025, 0.05, 0.1, 0.2, 0.3, 0.4, 0.5, 0.75, 1 mM), followed by incubation at 37 °C in a thermostatic water bath for 50 min, after which fluorescence intensity was detected using a fluorescence spectrophotometer.

Observation of fluorescence changes of MTP-NO_2 NPs and NTP-NO_2 NPs in 4T1/HUVEC cells under different treatments: 4T1/HUVEC cells were inoculated into glass-bottomed dishes at a density of 1×10^5 cells/dish and incubated overnight for cell attachment. Subsequently, cells were treated with GSH (0.2 mM, added 2 h in advance) and NEM (0.05 mM, added 1 h in advance) respectively, then co-incubated with MTP-NO_2 NPs or NTP-NO_2 NPs at a concentration of 10 μg/mL for 4 h. After that, the medium was discarded, cells were washed with PBS three times, and fluorescence images were acquired under CLSM.

Changes in absorption spectra of NTP-NO_2 NPs or MTP-NO_2 NPs after co-incubation with GSH

MTP-NO_2 NPs or NTP-NO_2 NPs (NPs concentration = 10 μg/mL) were mixed with GSH (0.2 mM) in a mixed solution of PBS:DMSO = 3:1 (v/v), incubated at 37 °C for 50 min, and the changes in nanoparticle absorption spectra were then recorded using a UV spectrophotometer.

In vitro cellular uptake

Cellular uptake of PS NPs was analyzed by CLSM. 4T1 cells were seeded in glass-bottom confocal dishes, cultured overnight (37 °C, 5% CO_2), then treated with DMEM containing MTP NPs, NTP NPs, MTP-NO_2 NPs, or NTP-NO_2 NPs (10 μg/mL). After incubation for 0, 1, 2, 4, 8 and 12 h, the cells were washed with PBS, followed by staining with Hoechst 33342 for 5 min, and imaged by CLSM.

Intracellular ROS detection

4T1 cells were seeded in glass bottom confocal dishes and cultured overnight at 37 ℃ in a humidified atmosphere containing 5% CO_2. The cells were then treated with 10 μg/mL MTP NPs, NTP NPs, MTP-NO_2 NPs, and NTP-NO_2 NPs, respectively. After 4 h of incubation, all cells were washed with PBS and treated with DCFH-DA (1 μM) for 20 min. After that, the cells in the light group were irradiated with 530 nm laser at 100 mW/cm^2 for 10 min, and the rest of the cells were incubated in the dark. Cell fluorescence was observed by CLSM to evaluate intracellular ROS production.

Live-dead cell staining

In order to visualize the anticancer effect of PDT, live/dead cell imaging was performed using a Calcein-AM/PI co-staining kit. Briefly, approximately 1×10^5 4T1 cells were inoculated into glass-bottomed petri dishes and cultured overnight at 37 °C under 5% CO_2 humidification. Then the cells were co-incubated with 60 μg/mL MTP NPs, NTP NPs, MTP-NO_2 NPs or NTP-NO_2 NPs for 4 h, then irradiated with a 530 nm (100 mW/cm^2) laser for 10 min, and then the cells were incubated for 12 h, the dark group was kept in the dark as a control. The cells were then stained with 1 μM Calcein-AM and 1 μM PI for 30 min and then residual dyes were washed out by PBS three times. Finally, Calcein-AM/PI fluorescence images were captured using CLSM to evaluate live and dead cells separately.

Cell viability

Cell viability was determined using the MTT assay, which is based on the reduction of 3-(4,5-dimethylthiazol-2-yl)-2,5-diphenyltetrazolium bromide (MTT) to formazan by mitochondrial succinate dehydrogenase. Briefly, 100 μL of cell suspension (5×10^3 cells/well) was added to a 96-well plate and incubated overnight at 37 °C in a humidified incubator containing 5% CO_2.

Then DMEM medium (100 μL) with different concentrations of nanoparticles (0-60 μg/mL) was added to the 96-well plate. After 4 h of incubation, the light group was irradiated with a 530 nm laser at an intensity of 100 mW/cm^2 for 10 min, while the dark group continued the incubation for 24 h without treatment. Subsequently, 20 μL of freshly prepared MTT solution (5 mg/mL) was added to each well, and aspirated after 4 h of incubation, followed by the addition of 150 μL of DMSO to dissolve the formazan crystals and the absorbance was measured by an enzyme counter at 490 nm.

Mitochondrial membrane potential assay

Rhodamine 123 was used as the mitochondrial membrane potential indicator. Briefly, approximately 1 × 10^5 4T1 cells were inoculated into glass-bottomed petri dishes and cultured overnight at 37 °C under 5% CO_2 humidification. Nanoparticles MTP NPs, NTP NPs, MTP-NO_2 NPs or NTP-NO_2 NPs (60 μg/mL) mixed with fresh medium were added to the dishes and incubated for 4 h. After 4 h of incubation, the light group was irradiated with a 530 nm laser (100 mW/cm^2, 10 min), in which the dark group was left without any other treatments, and the incubation was continued for 12 h. Subsequently, it was washed three times with PBS, and then with the membrane potential dye Afterwards, the cells were washed three times with PBS, then stained with Rhodamine 123 for 20 min, and stained with Hoechst 33342 for 5 min, and then photographed with confocal fluorescence imaging.

Co-localization imaging

4T1 cells were seeded in confocal-specific glass-bottom dishes and cultured overnight at 37 °C in a humidified atmosphere containing 5% CO_2. Subsequently, cells were incubated with fresh medium containing nanoparticles (5 μg/mL) for 4 h, washed three times with PBS, then stained respectively with commercial organelle dyes (Mito-Tracker Green for mitochondria, Lyso-Tracker Green for lysosomes, and Lipid-Green for lipids) for 20 min, and finally stained with Hoechst 33342 for nuclei. Subcellular localization of nanoparticles was observed via CLSM.

Transcriptome sequencing

Transcriptome sequencing was entrusted to Gene Denovo Biotechnology Co. (Guangzhou, China) for the services including library construction, sequencing, and bioinformatics analysis. Briefly, polyadenylated mRNA was enriched using mRNA Capture Beads, fragmented, and

reverse-transcribed into cDNA. After end-repair and adapter ligation, libraries were amplified by PCR and sequenced on the Illumina Novaseq X Plus platform. Raw reads were filtered by fastp to remove adapter-contaminated sequences, reads with > 10% undetermined bases, and low-quality reads (Q-score ≤ 20 in > 50% of bases). Clean reads were aligned to a ribosomal RNA database via Bowtie2 for rRNA depletion, followed by reference genome alignment using HISAT2. Gene expression was quantified as FPKM and TPM using StringTie and RSEM.

KEGG pathway enrichment analysis

Differentially expressed genes were annotated to KEGG pathways using the hypergeometric test. Significance was adjusted by the Benjamini-Hochberg method to control the false discovery rate (FDR), with FDR ≤ 0.05 set as the significance threshold. Functional mapping and statistical analysis were performed via the clusterProfiler R package. Enriched pathways were visualized in scatterplots or bar charts ranked by significance.

RBC hamolysis experiment

All animal studies according to the Guide for Care and Use of Laboratory Animals, approved by the Animal Experimentation Ethies Committee of Southern Medical University (00323972). Fresh whole blood was collected from healthy BALB/c mice (female, 3-5 weeks old) in an anti-coagulation tube and was isolated by centrifuging at 3000 rpm for 10 min to obtain red blood cells (RBCs). RBCs were washed with PBS buffer until the supernatant was colorless. RBCs were then adjusted to a concentration of 4% RBCs solutions by PBS buffer. Next, 0.5 mL of the above RBCs were mixed with 0.5 mL of nanoparticles suspension to obtain different concentrations ranging from 0 to 100 μg/mL, respectively. All of the above samples were put into a constant temperature shaker at 37 °C for 2 h. After the supernatant of each sample was collected through centrifuging at 3000 rpm for 10 min, 100 μL of the above supernatant sample was added to a 96-well plate. The absorption of the supernatant sample was measured at 540 nm using a microplate reader. To avoid the absorption influence of nanoparticles themselves, the absorption of the same concentration of nanoparticles without RBCs was measured to act as the negative control. A 0.5 mL 2% Triton X-100 PBS buffer (red blood cell lysis buffer) was mixed with 0.5 mL 4% RBCs to act as a positive control. The Hemolysis rate was calculated by the following equation:

$$\text{Hemolysis (\%)} = (A_{sam} - A_{neg}) / A_{pos} \times 100\%$$

A_{sam} is the absorption of sample (nanoparticles + RBCs); A_{neg} is the absorption of negative control (only nanoparticles); A_{pos} is positive control (nanoparticles + Triton X-100).

Blood clearance

Healthy BALB/c mice (female, 4-6 weeks old) were randomly grouped (n = 3) and administered nanoparticles NTP NPs or NTP-NO_2 NPs (6 mg/kg, 100 μL) via tail vein injection. At 0, 1, 2, 4, and 8 h post-injection, 200 μL of orbital venous blood was collected using heparin sodium as anticoagulant. Plasma was obtained by centrifugation at 4 °C and 3000 rpm for 10 min, diluted 20-fold with PBS buffer, and its fluorescence intensity was measured using a fluorescence spectrophotometer.

4T1 breast tumor-bearing BALB/c mice model

The tumor model was established by subcutaneously injecting 4T1 cells onto the right hind leg of female BALB/c mice, 4-6 weeks of age. Briefly, 1×10^6 4T1 cells were injected subcutaneously into the selected position. When the tumor volume reached approximately 100 mm^3, the mice were used for various *in vivo* studies.

Antitumor study *in vivo*

The obtained 4T1 tumor-bearing mice were randomly divided into 6 groups and there were 6 mice in each group. When the tumors grew to about 100 mm^3, the mice were intravenously injected with PBS or NTP NPs or NTP-NO_2 NPs (6 mg/kg, 100 μL) in different treatment groups. After 4 h, the mice in light groups were exposed to 530 nm laser for 12 min at 200 mW/cm^2. The body weight and tumor volume were monitored every two days. After 16 days, the heart, liver, spleen, lung, kidney, and tumor tissues were harvested for H&E, Tunel, Ki67 staining.

***In vivo* antimetastasis study**

On day -7, 4T1 cells were subcutaneously injected into the right hind limb of female BALB/c mice to establish the primary tumor-bearing model. On day -3, 5×10^5 4T1 cells were injected via the tail vein to establish the lung metastasis model. The treatment groups and methods were consistent with those in the *in vivo* antitumor experiment. On day 16 of the experiment, all animals were euthanized humanely according to standard protocols. The lung tissues were

collected, fixed with Bouin's fixative for 12 h to observe surface nodules, and subjected to H&E staining for histological examination of pulmonary nodules.

Statistical analysis

The statistical analyses were performed using GraphPad Prism 10.4.0 software. The statistical significance was assessed using one-way ANOVA. A p value ≥ 0.05 indicates no statistically significant difference, while $*p < 0.05$, $**p < 0.01$, $***p < 0.001$ and $****p < 0.0001$ denote statistically significant differences between the two groups being compared.

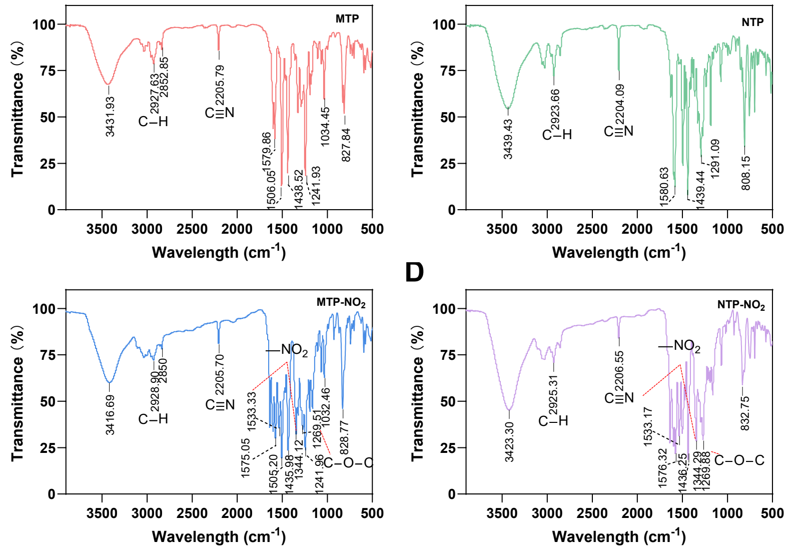

Figure S15. FT-IR spectra of (A) MTP, (B) NTP, (C) MTP-NO_2, and (D) NTP-NO_2.

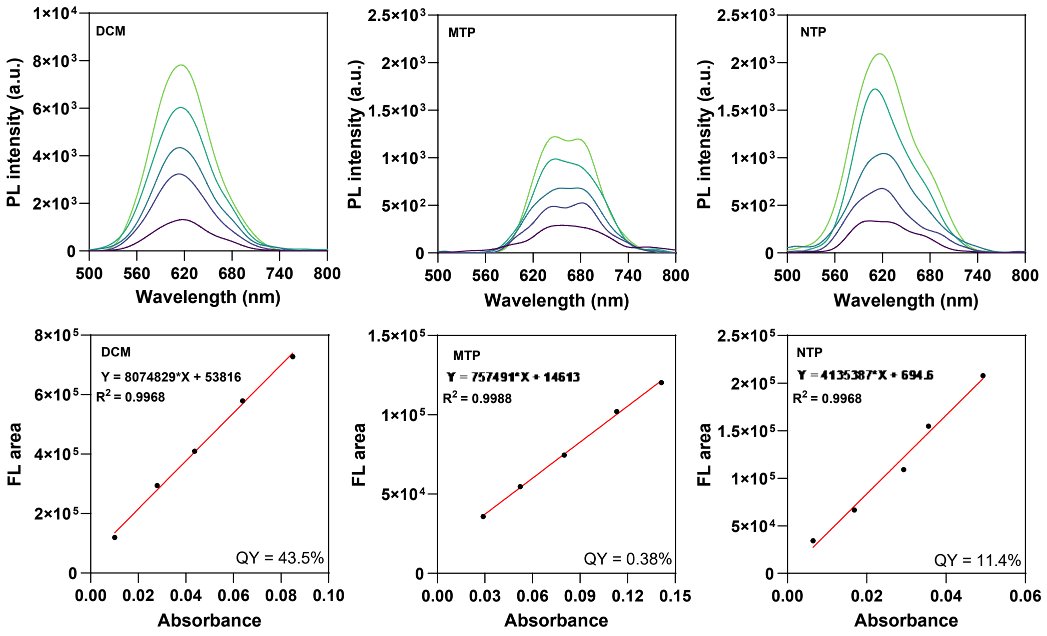

Figure S16. Fluorescence spectra of (A) DCM with different absorbance values in methanol, (B) MTP and (C) NTP measured in aqueous solution, along with linear fitting of fluorescence intensity integration versus absorbance (used for calculating fluorescence quantum yields).

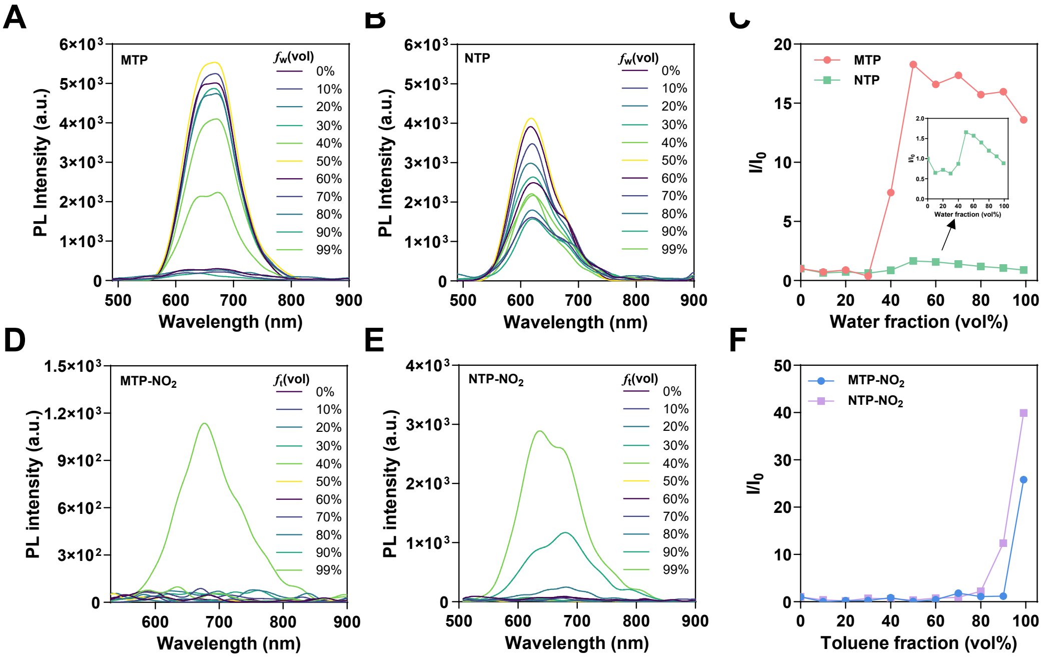

Figure S17. (A) MTP and (B) NTP fluorescence spectra in DMSO/water mixtures with varying water fractions (f_w). Excitation wavelengths: λ_{ex} = 455 nm for MTP and λ_{ex} = 470 nm for NTP. (C) Relative fluorescence intensities (I/I_0) of MTP and NTP at their maximum emission wavelengths as a function of f_w, where I_0 and I represent fluorescence intensities at f_w = 0% and f_w = 0-99%, respectively. (D) MTP-NO_2 and (E) NTP-NO_2 fluorescence spectra in DMSO/toluene mixtures with varying toluene fractions (f_t). Excitation wavelengths: λ_{ex} = 487 nm for MTP-NO_2 and λ_{ex} = 506 nm for NTP-NO_2. (F) Relative fluorescence intensities (I/I_0) of MTP-NO_2 and NTP-NO_2 at their maximum emission wavelengths as a function of f_t, where I_0 and I represent fluorescence intensities at f_t = 0% and f_t = 0-99%, respectively.

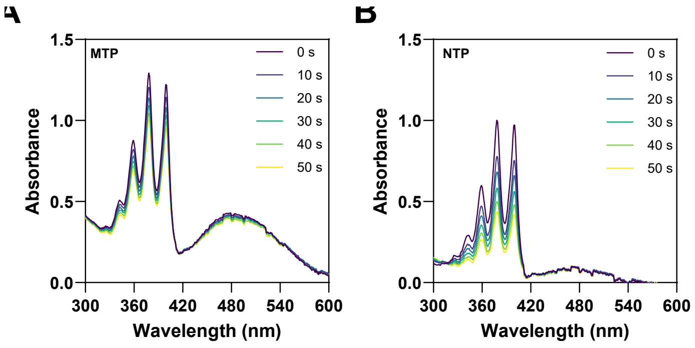

Figure S18. Decomposition rate of ABDA (100 μM) induced by $^1O_2$ generation in the following groups: (A) MTP, (B) NTP. (AIE PSs = 10 μg/mL, laser = 530 nm, 100 mW/cm^2).

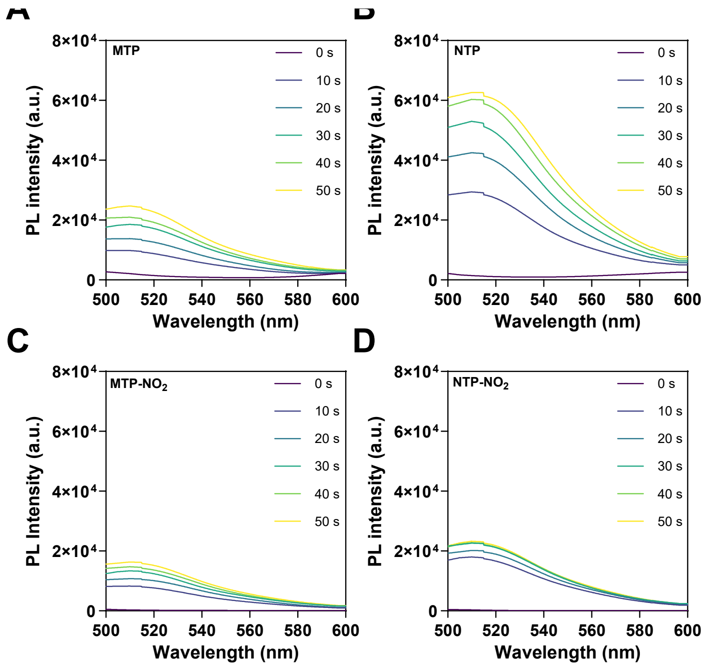

Figure S19. Fluorescence spectra of the mixed solution of HPF (5×10^{-6} M) in DMSO : water = 1:99 with (A) MTP, (B) NTP, (C) MTP-NO_2, and (D) NTP-NO_2 (the concentration of all AIE PSs = 10 μg/mL) under irradiation (530 nm, 100 mW/cm^2) with different time.

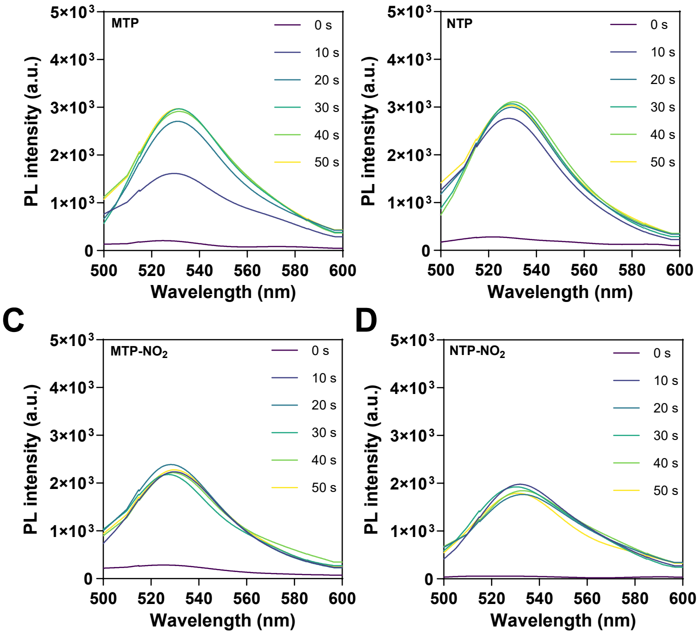

Figure S20. Fluorescence spectra of the mixed solution of H2DCF (2×10^{-5} M) in DMSO : water = 1:99 with (A) MTP, (B) NTP, (C) MTP-NO_2, and (D) NTP-NO_2 (the concentration of all AIE PSs = 10 μg/mL) under irradiation (530 nm, 100 mW/cm^2) with different time.

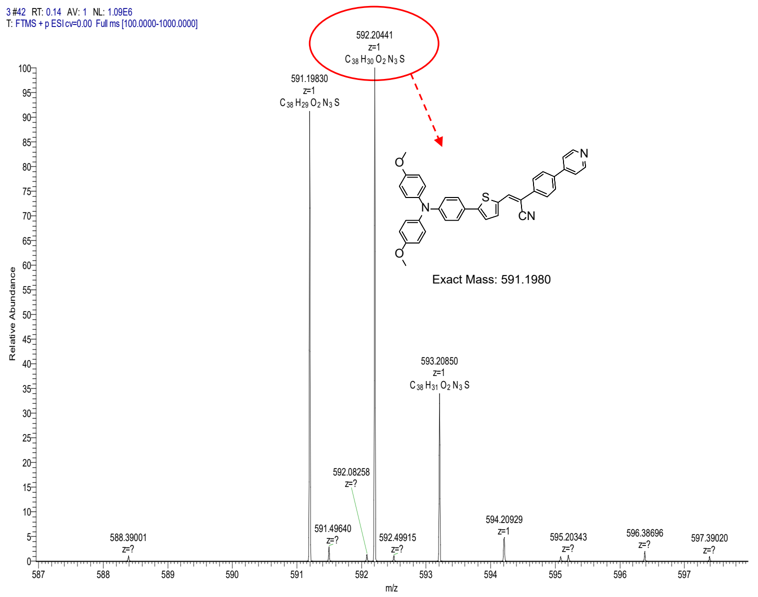

Figure S21. Mass spectrum of MTP.

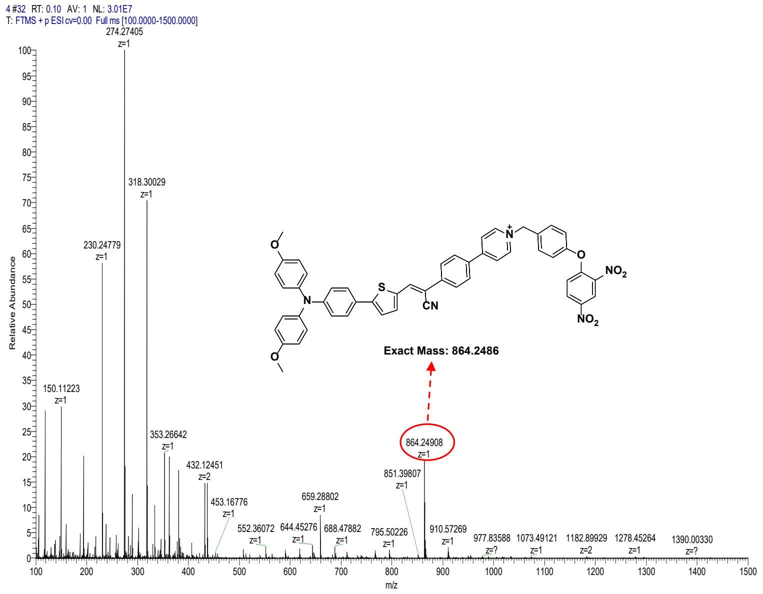

Figure S22. Mass spectrum of MTP-NO_2.

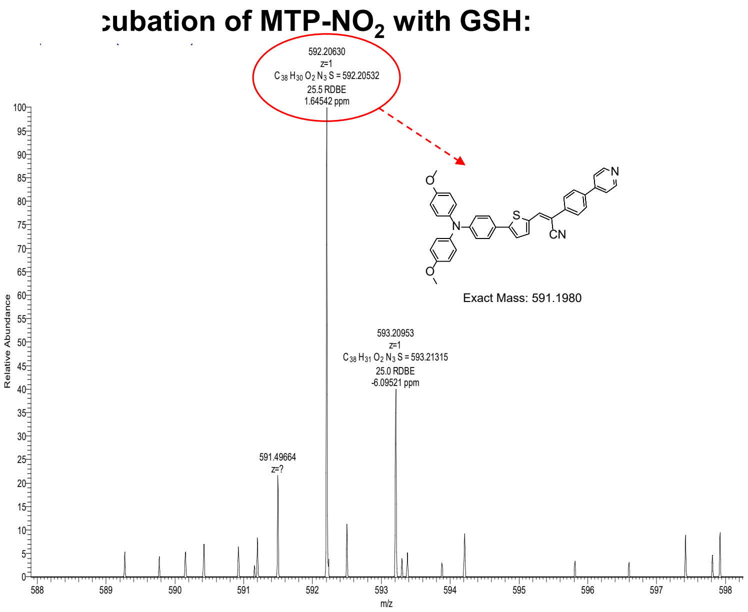

Figure S23. Mass spectrum of MTP-NO_2 after incubate with GSH.

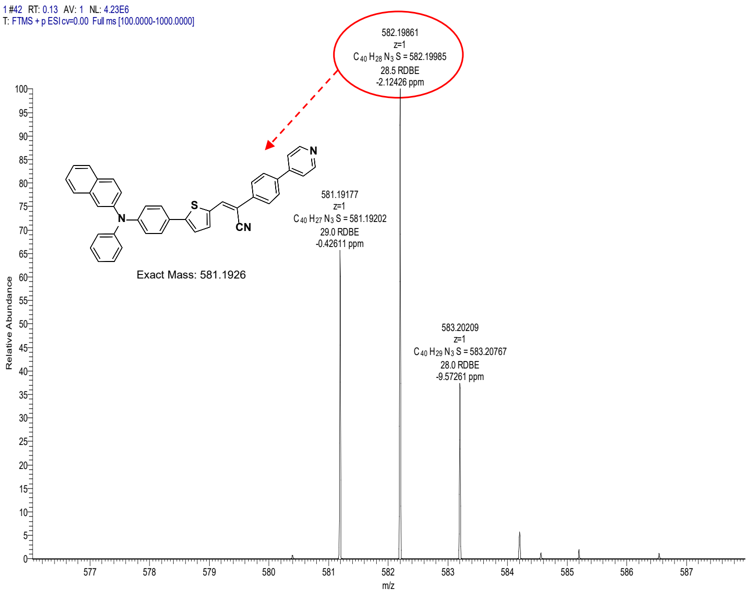

Figure S24. Mass spectrum of NTP.

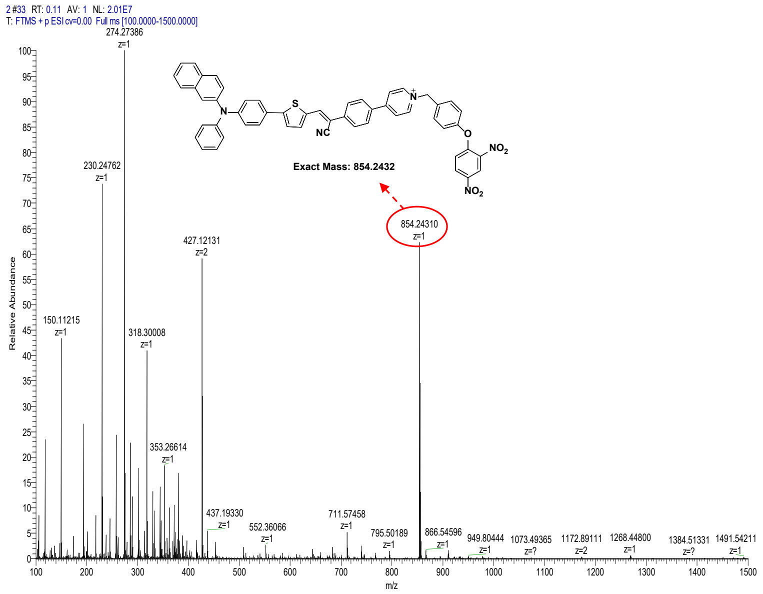

Figure S25. Mass spectrum of NTP-NO_2.

Co-incubation of NTP-NO_2 with GSH:

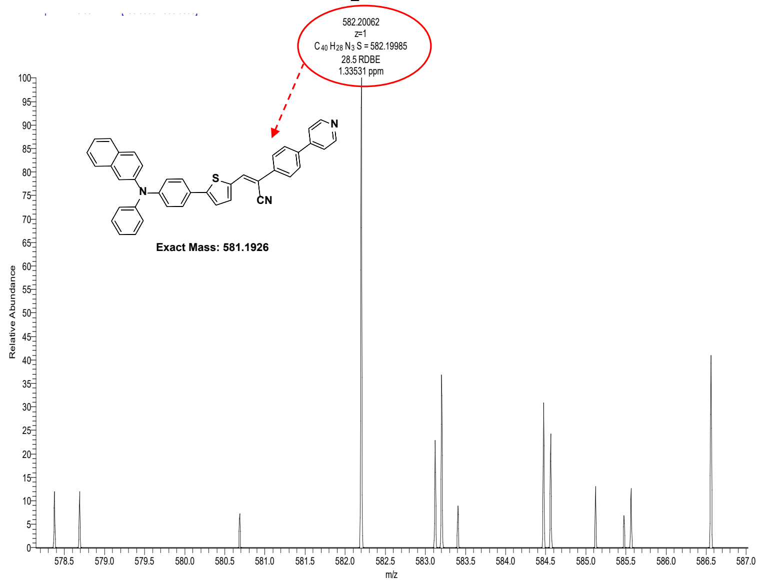

Figure S26. Mass spectrum of NTP-NO_2 after co-incubation with GSH.

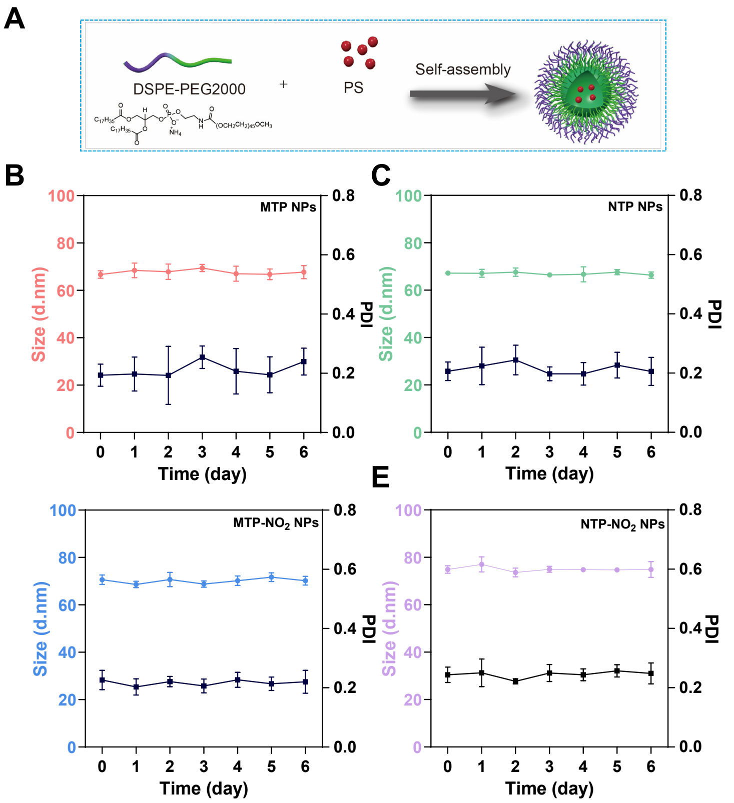

Figure S27. (A) Schematic diagram of nanoparticle synthesis. The hydrodynamic size changes of (B) MTP NPs, (C) NTP NPs, (D) MTP-NO_2 NPs and (E) NTP-NO_2 NPs within 6 consecutive days, measured by DLS (n = 5). Error bars represent the mean ± SD.

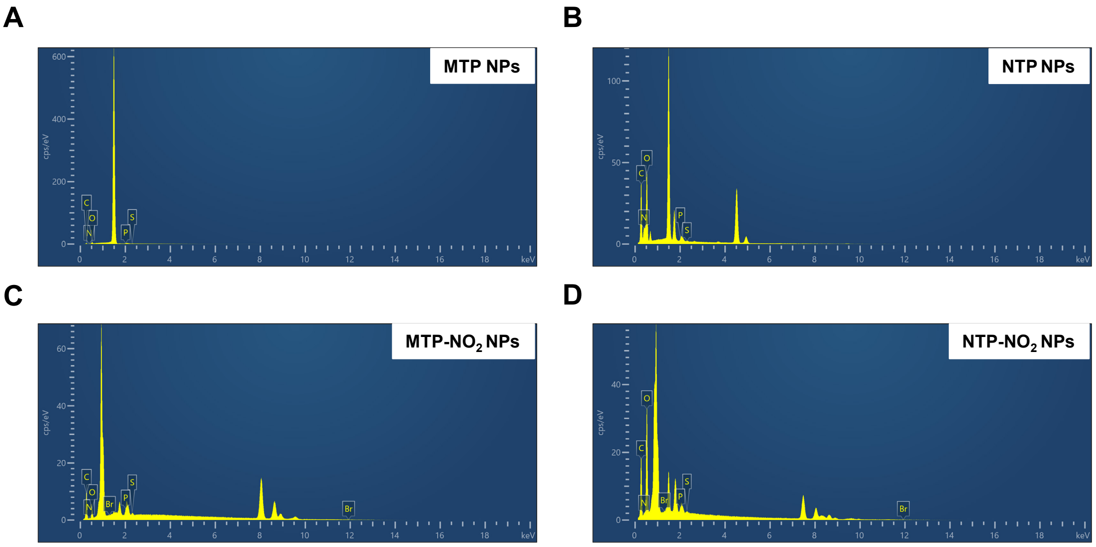

Figure S28. EDS spectra on the main element contents of (A) MTP NPs, (B) NTP NPs, (C) MTP-NO_2 NPs, and (D) NTP-NO_2 NPs.

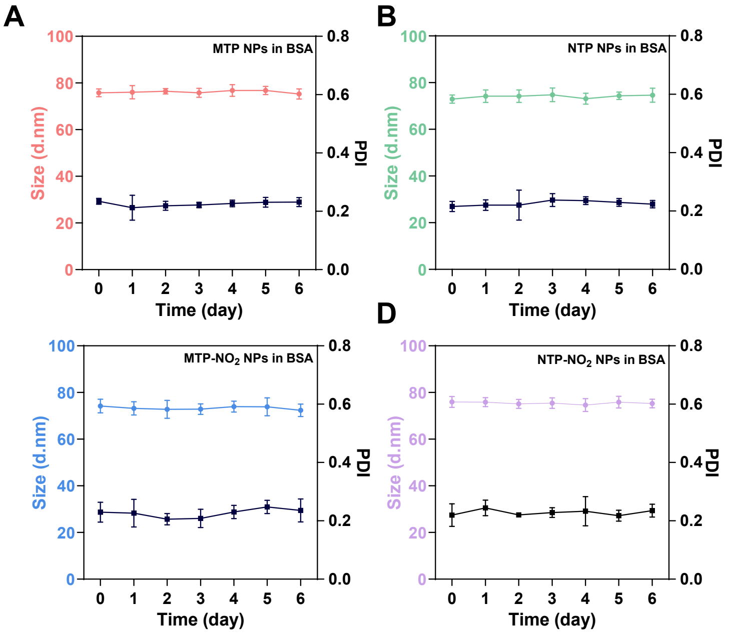

Figure S29. The hydrodynamic size changes of (A) MTP NPs, (B) NTP NPs, (C) MTP-NO_2 NPs, and (D) NTP-NO_2 NPs in an aqueous solution containing 10% BSA for 6 consecutive days, measured by DLS (n = 5). Error bars represent the mean ± SD.

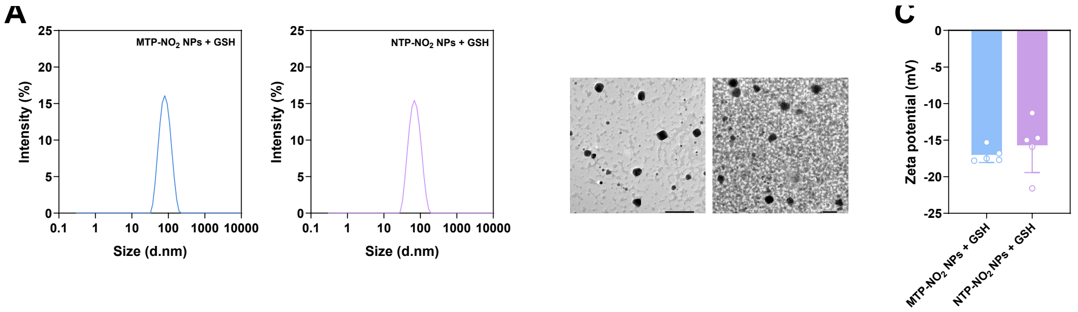

Figure S30. (A) DLS size distribution profiles, (B) TEM images, and (C) zeta potentials of MTP-NO_2 NPs and NTP-NO_2 NPs after incubation with GSH.

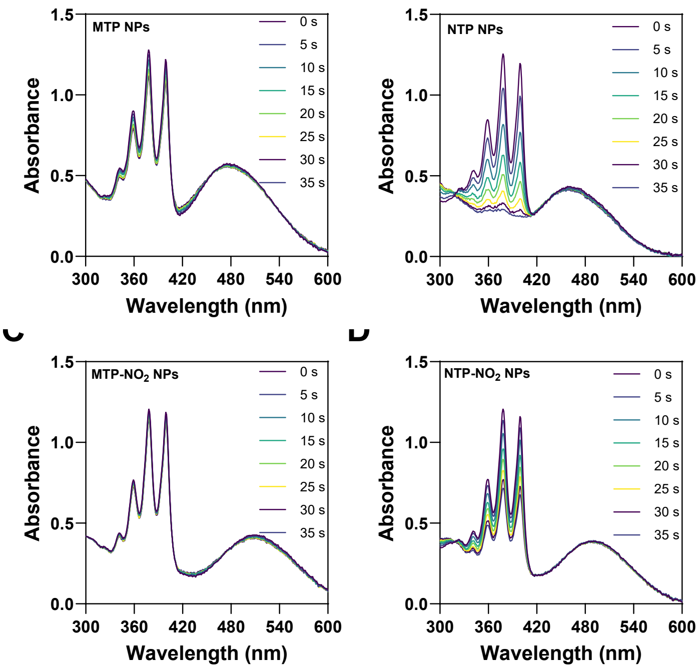

Figure S31. Absorption spectra of the mixed aqueous solution of ABDA (100 μM) with (A) MTP NPs, (B) NTP NPs, (C) MTP-NO$_2$ NPs, and (D) NTP-NO$_2$ NPs under different time with 100 mW/cm^2 530 nm laser irradiation. (the concentration of all nanoparticles = 10 μg/mL).

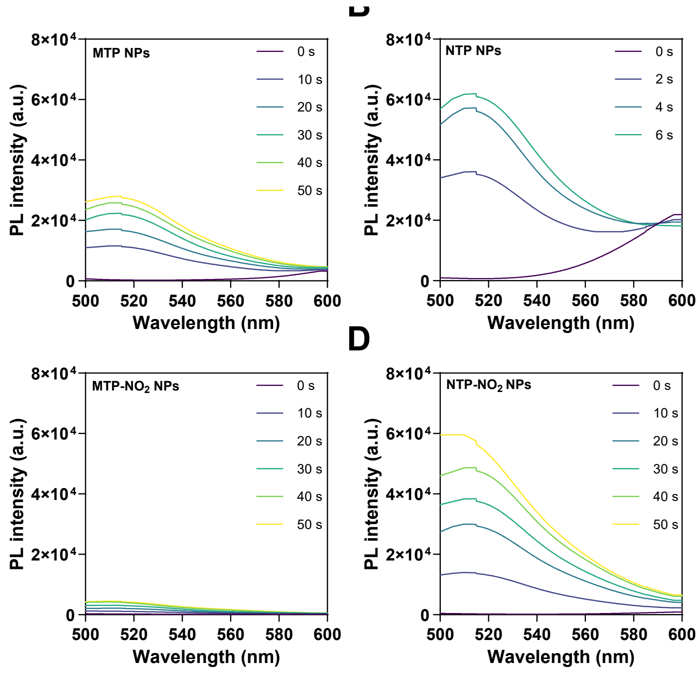

Figure S32. Fluorescence spectra of the mixed aqueous solution of HPF (5×10^{-6} M) with (A) MTP NPs, (B) NTP NPs, (C) MTP-NO_2 NPs, and (D) NTP-NO_2 NPs in water under irradiation (530 nm, 100 mW/cm^2). (the concentration of all nanoparticles = 10 μg/mL).

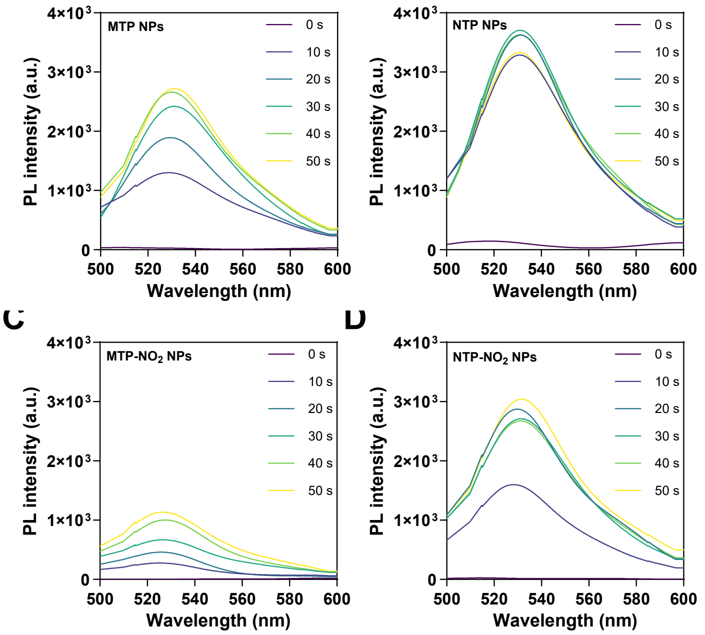

Figure S33. The fluorescence spectra of H2DCF (2×10^{-5} M) after irradiation (530 nm, 100 mW/cm^2) for different time in the presence of 10 μg/mL (A) MTP NPs, (B) NTP NPs, (C) MTP-NO_2 NPs, and (D) NTP-NO_2 NPs in water.

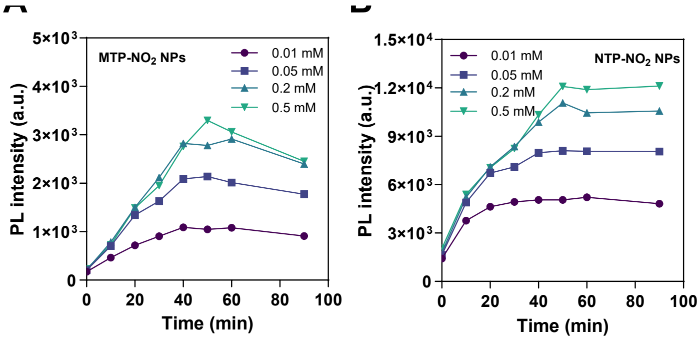

Figure S34. The fluorescence intensity of (A) MTP-NO_2 NPs and (B) NTP-NO_2 NPs (10 μg/mL) with different concentrations of GSH (0.01 mM, 0.05 mM, 0.2 mM, 0.5 mM) versus the reaction time.

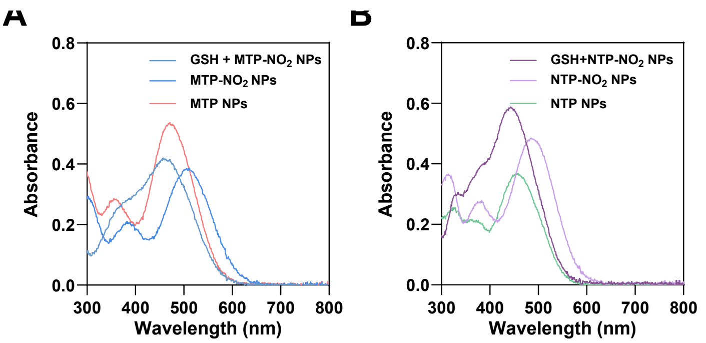

Figure S35. (A) UV-vis absorption spectra of MTP-NO_2 NPs, MTP NPs, and GSH + MTP-NO_2 NPs in PBS/DMSO (v/v = 3/1) after co-incubation at 37 °C for 50 min. (B) UV-vis absorption spectra of NTP-NO_2 NPs, NTP NPs, and GSH + NTP-NO_2 NPs in PBS/DMSO (v/v = 3/1) after co-incubation at 37 °C for 50 min.

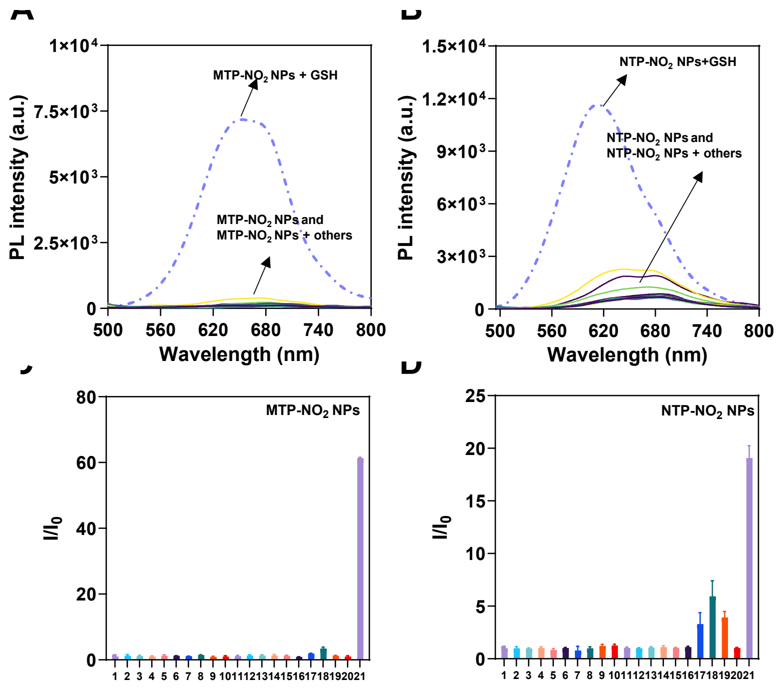

Figure S36. Fluorescence spectra of (A) MTP-NO_2 NPs and (B) NTP-NO_2 NPs (10 µg/mL) in PBS/DMSO (v/v = 3/1) aqueous buffer in the presence of GSH (0.2 mM) or other various substances (10 mM). Corresponding fluorescence histograms showing emission intensities at 615 nm wavelength for (C) MTP-NO_2 NPs and (D) NTP-NO_2 NPs after adding various acids, inorganic salts, and amino acids (n = 3). (1-K_3PO_4, 2-NaCl, 3-CH_3OOK, 4-Na_2SO_4, 5-CuI, 6-Vitamin C, 7-KOH, 8-CH_3CH_2ONa, 9-$FeCl_3$, 10-$AgCO_3$, 11-L-Phenylalanine, 12-Glutamate, 13-L-Lysine, 14-Glycine, 15-L-Alanine, 16-L-Valine, 17-L-Tyrosine, 18-NaOH, 19-$AlCl_3$, 20-Free, 21-GSH). Error bars represent the mean ± SD.

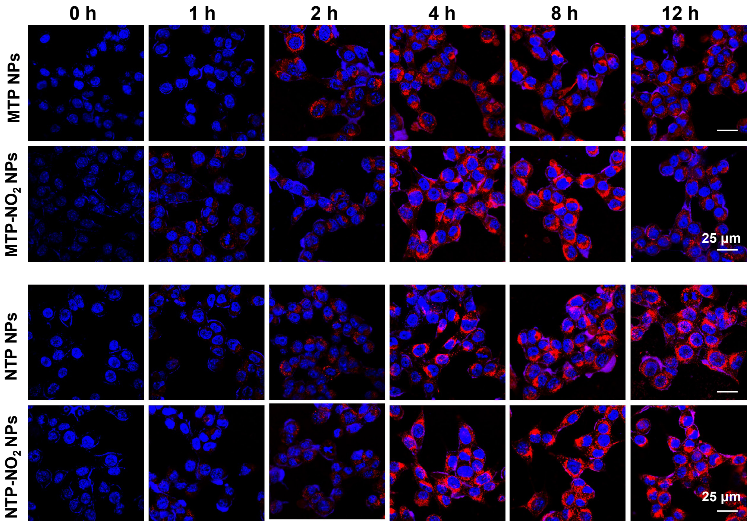

Figure S37. CLSM images of 4T1 cells after treatment with MTP NPs, MTP-NO_2 NPs, NTP NPs or NTP-NO_2 NPs (10 μg/mL) for 0, 1, 2, 4, 8 and 12 h.

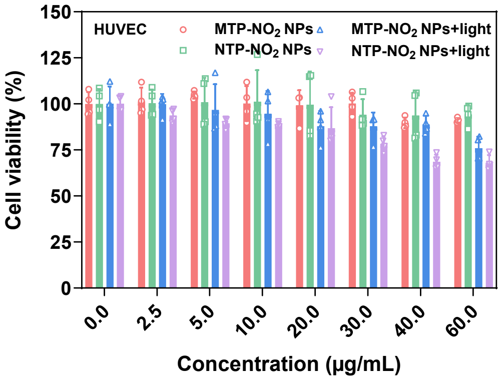

Figure S38. Effects of MTP-NO_2 NPs and NTP-NO_2 NPs at various concentrations on the cell viability of HUVECs with or without laser irradiation. Light irradiation conditions: 100 mW/cm^2 for 10 min.

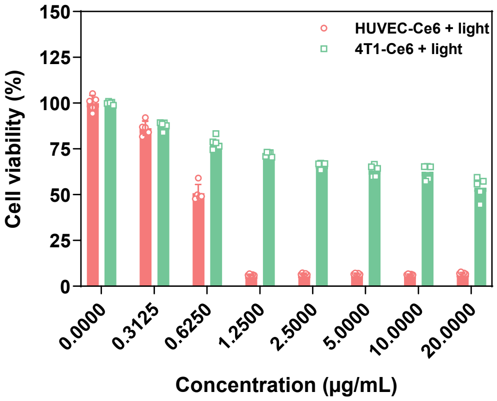

Figure S39. Cell viability of 4T1 cells and HUVECs treated with Ce6 at varying concentrations,

with or without light irradiation. [Irradiation intensity: 30 mW/cm^2; irradiation duration: 3 min]

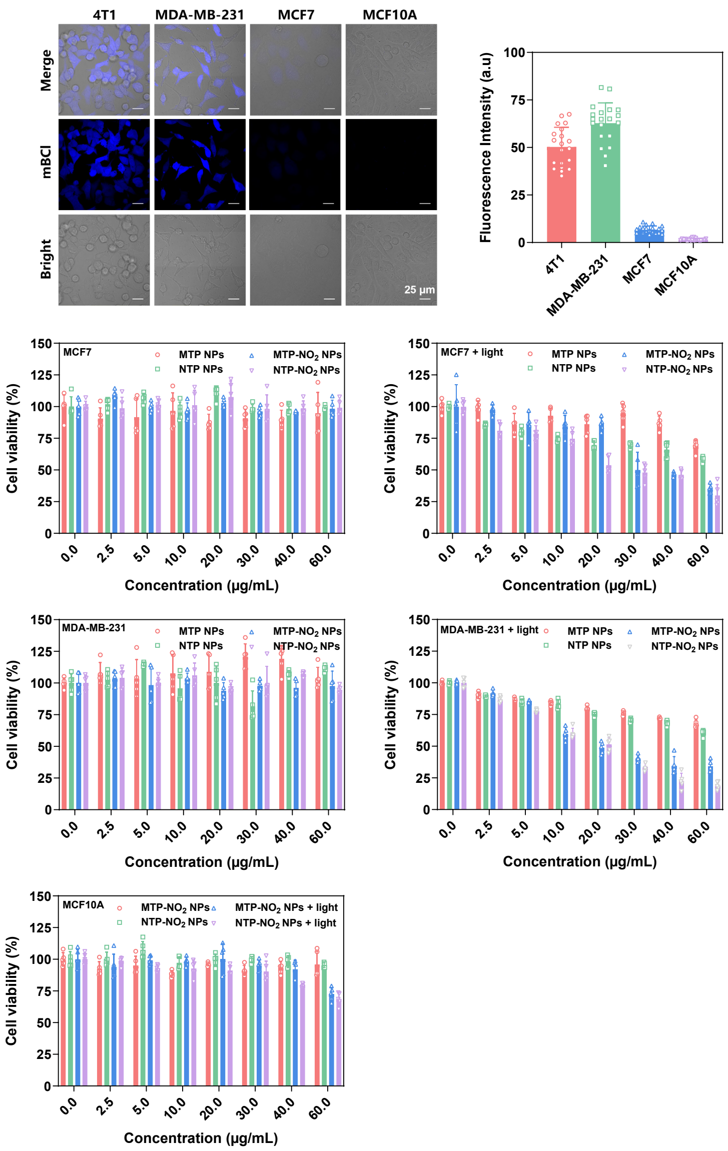

Figure S40. (A) CLSM images of 4T1, MDA-MB-231, MCF7 and MCF10A cells stained with mBCl. (B) The fluorescence quantitative intensity in (A). Cell viability of (C) MCF7 and (D) MDA-MB-231 cells treated with MTP NPs, NTP NPs, MTP-NO_2 NPs, or NTP-NO_2 NPs at varying concentrations, with or without light irradiation. (E) Cell viability of MCF10A cells treated with MTP-NO_2 NPs or NTP-NO_2 NPs at varying concentrations, with or without light irradiation. [Irradiation intensity: 100 mW/cm^2; irradiation duration: 10 min]

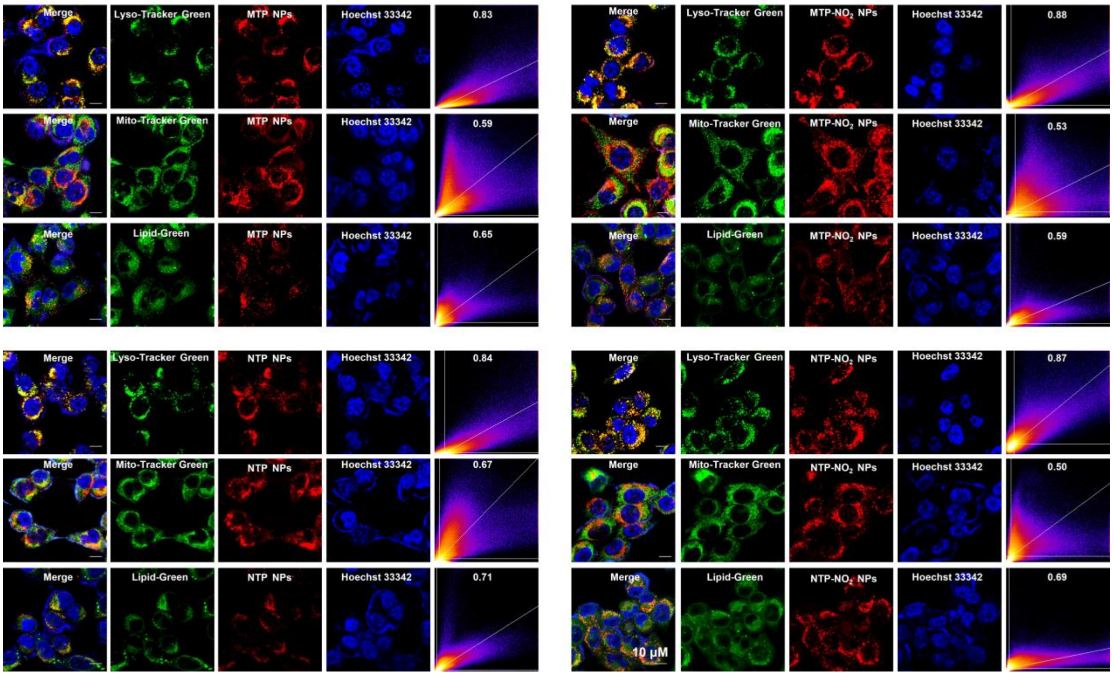

Figure S41. CLSM images of 4T1 cancer cells treated with MTP NPs, NTP NPs, MTP-NO_2 NPs or NTP-NO_2 NPs, which were co-stained with commercial Lyso-Tracker Green, Mito-Tracker Green or Lipid-Green.

Table S1. Cellular colocalization of different nanoparticles.

NPs	Lyso-Trancker Green	Mito-Tracker Green	Lipid-Green
MTP	0.83	0.59	0.65
NTP	0.84	0.67	0.71
MTP-NO_2	0.88	0.53	0.59
NTP-NO_2	0.87	0.50	0.69

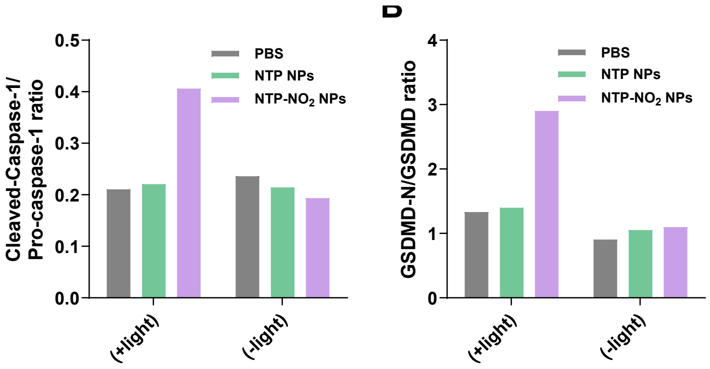

Figure S42. (A) The ratios of cleaved Caspase-1 to pro-Caspase-1 and (B) the ratios of GSDMD-N to GSDMD were statistically analyzed.

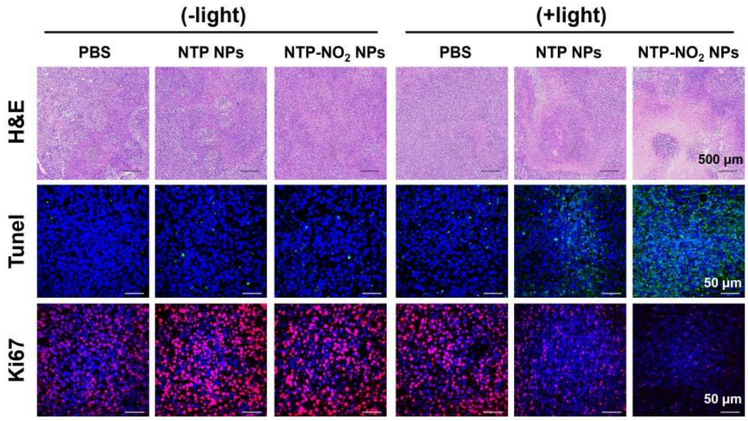

Figure S43. Representative images of H&E staining, TUNEL assay, and Ki67 immunohistochemical staining in tumor tissues after different treatments.

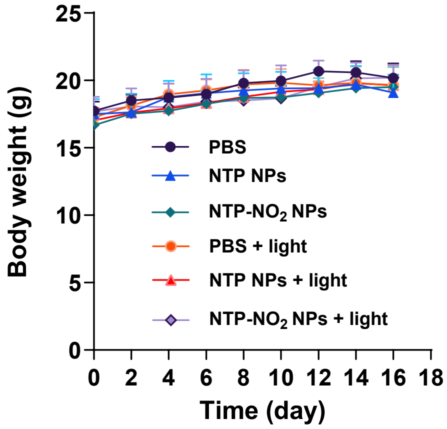

Figure S44. Body weight changes of mice in different treatment groups (n = 6). Error bars represent the mean ± SD.

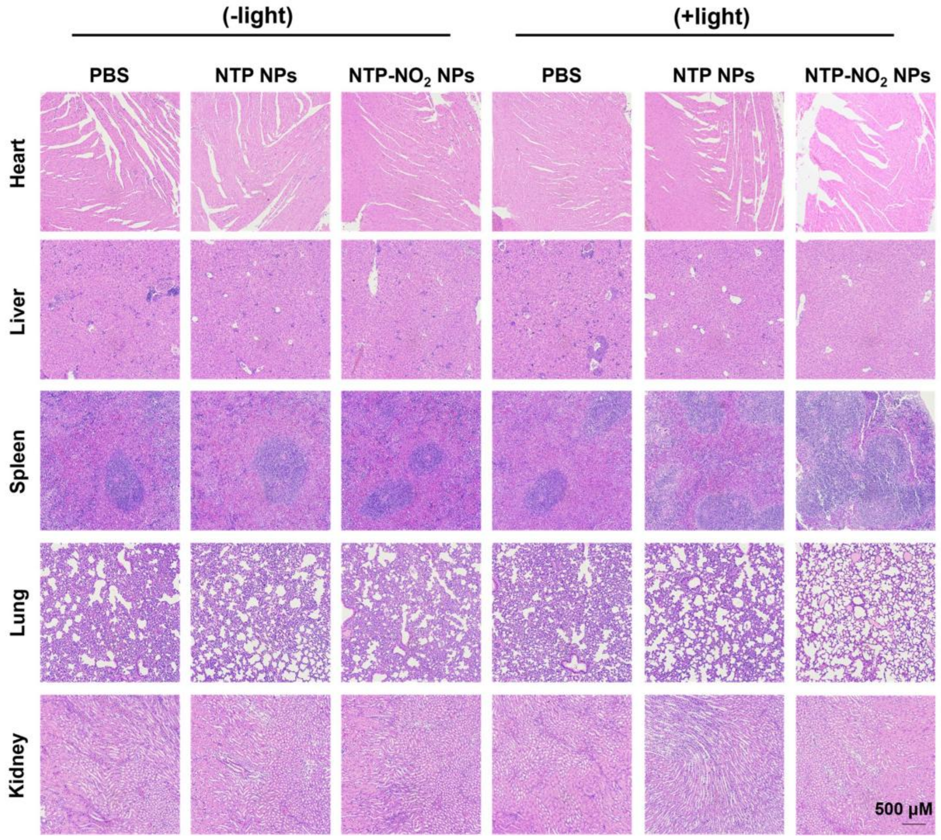

Figure S45. H&E staining results of heart, liver, spleen, lung, and kidney tissue sections from different groups of mice post-treatment.

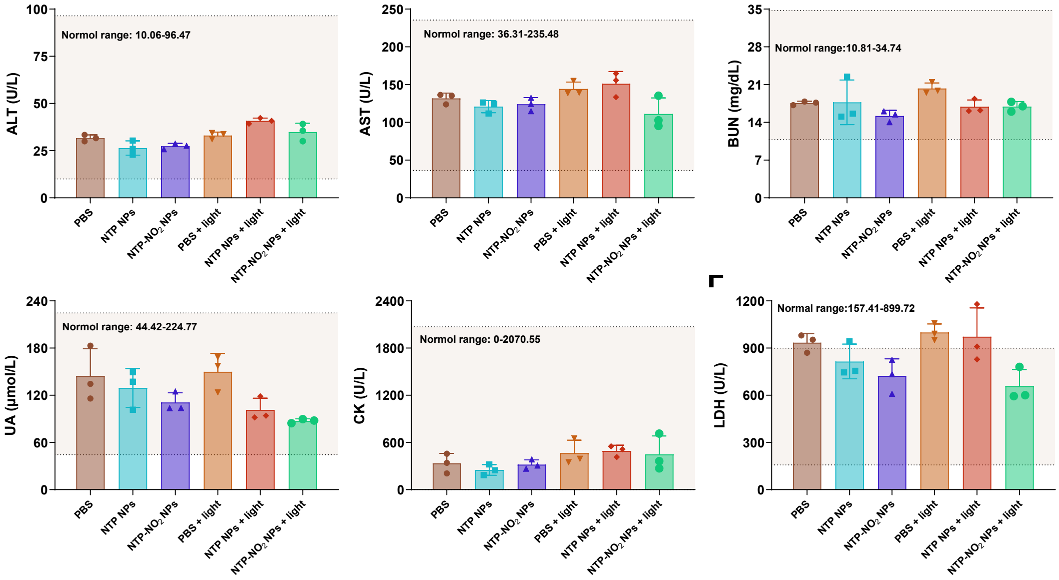

Figure S46. Blood biochemistry of mice on day 16: (A) alanine aminotransferase (ALT), (B) aspartate aminotransferase (AST), (C) blood urea nitrogen (BUN), (D) urea (UA), (E) creatine kinase (CK), (F) lactate dehydrogenase (LDH). Error bars represent the mean ± SD.

Table S2. The results of whole blood test for different groups. [a]

Test	Control	NTP NPs	$NTP-NO_2$ NPs	Control + light	NTP NPs + light	$NTP-NO_2$ NPs + light	Mice without tumor	Normal range
WBC	152.9±57.17	208.23±21.68	143.97±31.50	125.53±41.30	49.6±34.45	7.37±3.59	3.43±0.40	0.8-10.6
Lymph#	53.97±24.52	58.8±58.57	80.93±51.19	54.67±12.19	32.63±19.02	5.8±2.72	2.87±0.45	0.6-8.9
Mon#	7.57±4.75	9.8±1.56	7.1±1.5	5.57±2.69	3.13±3.96	0.23±0.23	0.1±0.00001	0.04-1.4
Gran#	91.37±73.27	139.73±71.18	55.93±26.54	65.3±49.45	13.83±18.80	1.33±0.67	0.47±0.06	0.23-3.6
Lymph%	44.03±36.00	29.07±29.72	52.83±28.76	50.07±30.78	73.03±22.45	78.87±2.28	84.07±3.29	40-92
Mon%	4.63±1.86	4.77±1.00	4.97±0.06	4.23±0.99	5.57±3.65	3.13±1.11	2.6±0.56	0.9-18
Gran%	51.33±35.13	66.17±30.64	42.2±28.74	45.7±29.80	21.4±18.98	18±1.51	13.33±2.73	6.5-50
RBC	7.94±1.01	7.61±0.36	8.44±0.23	8.02±0.23	7.9±0.20	6.05±3.90	8.53±0.58	6.5-11.5
HGB	135±10.82	127±2.65	138±1	129.33±6.81	127.33±6.03	102.67±57.87	136.67±8.74	110-165
HCT	38.33±5.46	35.67±1.40	42±0.72	39.07±1.98	38.37±0.75	29.6±19.40	43.3±2.43	35-55
MCV	48.33±10.1	46.97±0.60	49.8±0.53	48.73±1.18	48.63±1.16	48.27±1.67	50.9±0.79	41-55
MCH	17.07±0.91	16.67±0.55	16.33±0.55	16.07±0.47	16.03±0.67	18.57±3.85	16±0.1	13-18
MCHC	353.67±22.55	355.67±7.37	328.33±7.23	330.67±2.31	331.33±9.61	388.33±97	315±4.36	300-360
RDW	16±0.52	15.67±0.91	15.47±0.68	15.67±0.35	15±0.3	16.77±1.36	17.7±2.51	12.0-19.0
PLT	621.67±108.70	766±48.03	704.33±157.61	751.67±315.44	475.67±95.47	633.67±381.23	708±223.63	400-1600
MPV	5.77±0.21	6.53±0.25	6.53±0.21	6.1±0.3	6.03±0.59	6±0.89	5.47±0.21	4.0-6.2
PDW	16.8±0.3	16.67±0.40	17±0.1	17.27±0.29	17±0.2	17.1±0.36	16.6±0.3	12.0-17.5
PCT	0.357±0.06	0.50±0.05	0.46±0.09	0.46±0.21	0.29±0.09	0.41±0.26	0.38±0.11	0.100-0.780

[a] White blood cell systems: WBC, Lymph#, Mon#, Gran#, Lymph%, Mon%, Gran%; red blood cell systems: RBC, HGB, HCT, MCV, MCH, MCHC, RDW; blood platelet systems: PLT, MPV.

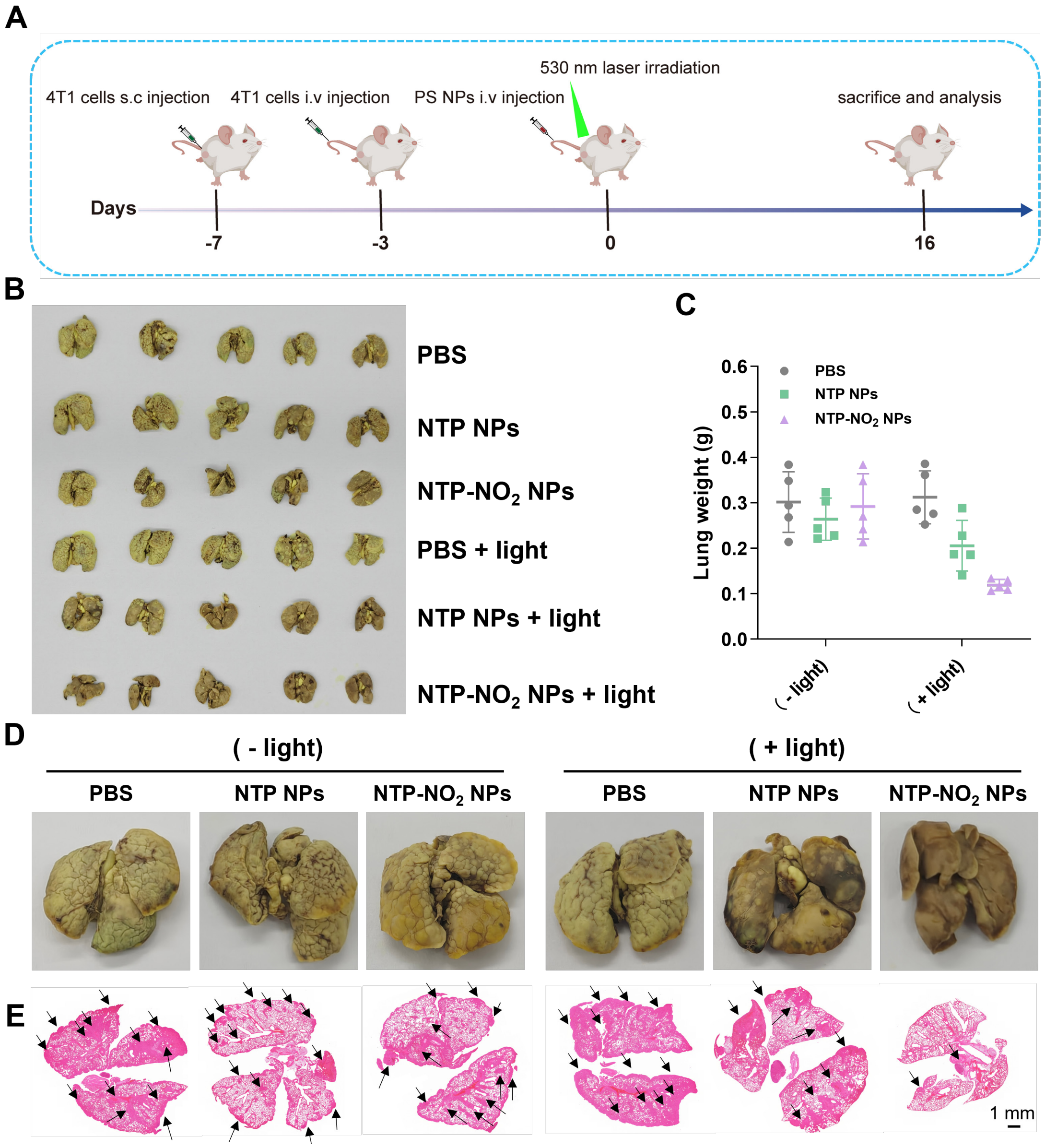

Figure S47. (A) Schematic illustration of the establishment protocol and treatment timeline for the 4T1 breast cancer lung metastasis model. (B) Excised mouse lung specimens from the PBS, NTP NPs, NTP-NO_2 NPs, PBS + light, NTP NPs + light, and NTP-NO_2 NPs + light groups on day 16, post-fixation with Bouin's solution (n = 5). (C) Statistical analysis of lung weights from treated mice in each group (n = 5). (D) Representative images of lung tissues from each group. (E) Histological images of whole lung lobes from each group stained with H&E, alongside their corresponding magnified views. Black arrows indicate grossly visible lung lesions. Error bars represent the mean ± SD.
